# Neuropathological changes and cognitive deficits in rats transgenic for human mutant tau recapitulate human tauopathy

**DOI:** 10.1016/j.nbd.2019.03.018

**Published:** 2019-07

**Authors:** Janice C. Malcolm, Lionel Breuillaud, Sonia Do Carmo, Hélène Hall, Lindsay A. Welikovitch, Jennifer A. Macdonald, Michel Goedert, A. Claudio Cuello

**Affiliations:** a Department of Anatomy and Cell Biology, McGill University, Montréal, QC H3A 0C7, Canada; b Department of Pharmacology and Therapeutics, McGill University, Montréal, QC H3G 1Y6, Canada; c Department of Neurology and Neurosurgery, McGill University, Montréal, QC H3A 2B4, Canada; d Medical Research Council Laboratory of Molecular Biology, Cambridge CB2 0QH, UK

**Keywords:** Alzheimer's disease, Tauopathy, Rat model, FTDP-17T, Inflammation, Neurodegeneration, Gliosis, Neuronal loss, AD, Alzheimer's disease, BSA, Bovine serum albumin, CaMKIIα, Calcium/calmodulin-dependent protein kinase II alpha subunit, CNS, Central nervous system, EM, Electron microscopy, EDTA, Ethylenediaminetetraacetic acid, EGTA, Ethylene glycol-bis(β-aminoethyl ether)-N,N,N′,N′-tetraacetic acid, FTDP-17T, Frontotemporal dementia and parkinsonism linked to chromosome 17 caused by *MAPT* mutations, GFAP, Glial Acidic Fibrillary Protein, HRP, Horseradish peroxidase, Iba1, Ionized calcium-binding adapter molecule 1, MAPT, Microtubule-associated protein tau, MRI, Magnetic resonance imaging, NFT, Neurofibrillary tangle, NOL, Novel Object Location, NOR, Novel Object Recognition, PBS, Phosphate buffered saline, PCR, Polymerase chain reaction, PFA, Paraformaldehyde, PMSF, Phenylmethylsulfonyl fluoride, TBS, Tris buffered saline

## Abstract

The assembly of tau protein into abnormal filaments and brain cell degeneration are characteristic of a number of human neurodegenerative diseases, including Alzheimer's disease and frontotemporal dementia and parkinsonism linked to chromosome 17. Several murine models have been generated to better understand the mechanisms contributing to tau assembly and neurodegeneration. Taking advantage of the more elaborate central nervous system and higher cognitive abilities of the rat, we generated a model expressing the longest human tau isoform (2N4R) with the P301S mutation. This transgenic rat line, R962-hTau, exhibits the main features of human tauopathies, such as: age-dependent increase in inclusions comprised of aggregated-tau, neuronal loss, global neurodegeneration as reflected by brain atrophy and ventricular dilation, alterations in astrocytic and microglial morphology, and myelin loss. In addition, substantial deficits across multiple memory and learning paradigms, including novel object recognition, fear conditioning and Morris water maze tasks, were observed at the time of advanced tauopathy. These results support the concept that progressive tauopathy correlates with brain atrophy and cognitive impairment.

## Highlights

• A transgenic rat model expressing the full length human tau with the P301S mutation.
• Progressive human-like tau brain pathology with recruitment of endogenous rodent tau.
• Pathology-induced gliosis, myelin breakdown, neuronal loss and ventricular dilation.
• Widespread behavioural deficits occur at end-stage tau pathology.

## 1 Introduction

Tau is a microtubule-associated protein that promotes microtubule assembly and, perhaps, stabilisation (Goedert, 2018; Weingarten et al., 1975). The aggregation of tau into abnormal filamentous inclusions underlies many neurodegenerative diseases. Six tau isoforms are expressed in the normal adult human brain: three isoforms have four microtubule-binding repeats (R1, R2, R3, R4; 4R tau) and three isoforms lack the second repeat (3R tau) (Goedert et al., 1989). In Alzheimer's disease (AD), pathological tau filaments are composed of all six brain isoforms (Goedert et al., 1992). The identification of disease-causing mutations in *MAPT*, the gene encoding tau, in cases of frontotemporal dementia established that dysfunction of tau protein is sufficient to cause neurodegeneration and dementia (Hutton et al., 1998; Poorkaj et al., 1998; Spillantini et al., 1998).

The generation of transgenic models expressing mutated human tau has enabled important insights into tauopathies (Goedert et al., 2012). Models of tauopathy have been generated in several species, including *Drosophila* (Chatterjee et al., 2008; Feuillette et al., 2010; Steinhilb et al., 2007), *C. elegans* (Kraemer et al., 2003; Miyasaka et al., 2005), zebrafish (Bai et al., 2007; Paquet et al., 2009) rat (Filipcik et al., 2012; Zilka et al., 2006) and, most commonly, mouse [for a review, see (Dujardin et al., 2015)].

We believe that there is a need to investigate tau pathology in additional models to gain a more comprehensive understanding of tau pathogenesis. Modelling tauopathies in rats has some major advantages over other species. Thus, the structure of rat ApoE protein resembles that of human ApoE4 (LaDu et al., 1997; Tran et al., 2013). This might be significant, since it has been shown that human ApoE expression exacerbates the progression of tau pathology in mice transgenic for human P301S tau (Shi et al., 2017). Additionally, rats have a more complex central nervous system (CNS), a richer behavioural repertoire and higher cognitive abilities compared with mice (Do Carmo and Cuello, 2013). Their larger brains also facilitate more in-depth investigation of novel biomarkers and experimental therapeutics (Parent et al., 2017; Zimmer et al., 2014).

To evaluate the capacity of rat transgenic models to recapitulate essential features of human tauopathy, we generated a transgenic line that expresses the 441 amino acid human tau isoform (2N4R) bearing the P301S mutation, driven by the CaMKIIα promoter. This promoter was chosen to limit pathology to brain regions associated with cognition and memory. The transgenic line, designated R962-hTau, developed numerous primary and secondary features associated with AD and other tauopathies, including filamentous tau inclusions, cognitive deficits, brain atrophy, ventricular dilation, neuronal loss, myelin degeneration and a progressive glial response. However, we would like to disclose that an unforeseen error in the breeding program resulted in the loss of the R962-hTau line.

## 2 Materials and methods

### 2.1 Generation of the R962-hTau transgenic line

The cDNA construct used to generate the transgenic rat contains a hybrid intron in the 5′-untranslated leader sequence, the coding region of the 2N4R isoform of human tau with the FTDP-17T P301S mutation (Bugiani et al., 1999), a potent Kozak sequence and a polyadenylation signal, under the control of the mouse CAMKIIα promoter cassette. hTauP301S was PCR-amplified from a plasmid containing human P301S 2N4R using mutated primers to change the Kozak sequence. The product was blunt-ended and inserted into the *Eco*RV site of the pNN265 intron plasmid. The hTauP301S cDNA with the intronic sequence were subcloned into the pMM403-CAMkinaseIIα-promoter plasmid.

Transgenic rats were generated by pronuclear injections of one-cell embryos (Wistar, Charles River Laboratories, Saint-Constant, Canada) at the Centre hospitalier de l'Université de Montréal Research Centre's transgenesis and animal modelling core facility. Founders were identified by PCR analysis of genomic DNA obtained from ear biopsy, using the primer pair SeqCamK2aForw 5’ CACTCAGGAGCACGGGCAGG 3′; Inter3-tau 5’ GCCTGGAGGGGCTGCTCCC 3′ (IDT, USA). A second, lower expresser line, designated R955-hTau, was also generated and is undergoing characterisation.

### 2.2 Animals

All animal work was approved by the McGill Animal Care Committee, under strict adherence to the guidelines set out by the Canadian Council on Animal Care. Heterozygous male and female R962-hTau transgenic rats and their wild-type (WT) littermates were used. They were housed in temperature- and humidity-controlled rooms with a 12 h light cycle, food and water *ad libitum.* We examined cohorts at 3, 6, 10, 15 and 18–20 months of age. Electron microscopy (EM) experiments were carried out using 12- and 21-month old rats.

### 2.3 Tissue preparation

Brain tissues were processed as previously described (Iulita et al., 2017). Rats were deeply anaesthetized, perfused transcardially with saline and their brains removed. One hemisphere was dissected, snap frozen and stored at −80 °C. The other hemisphere was post-fixed in 4% paraformaldehyde (PFA), saturated in 30% sucrose solution and sectioned using a freezing sledge microtome (Leica, SM 2000R; Germany).

### 2.4 Immunohistochemistry

Immunohistochemistry was performed on tissue from R962-hTau and control rats at 3, 6, 10, 15 and 18–20 months of age. For immunoenzymatic labelling, sections from each animal were quenched for 30 min in 3% H_2_0_2_ and 10% methanol in TBS, then blocked in 10% normal goat serum (NGS) in TBS containing 0.1% Tween (TBS-T) for 1 h. Sections were incubated with primary antibodies (Supplementary Table 1) at 4 °C overnight in 10% NGS in TBS. The following day, sections were incubated with either secondary goat-anti mouse (1:200) or biotinylated goat-anti rabbit IgG (1:200) for 1 h, then incubated with mouse anti-horseradish peroxidase (1:30) and horseradish peroxidase (HRP) [5 μg/ml (1:200)] (MAP kit, Medimabs, Canada) or Vectastain Elite ABC-HRP Kit (Vector Labs, USA) for 1 h. Finally, stainings were developed using 0.05% 3,3′-diaminobenzidine as the chromogen (Sigma-Aldrich, Germany) and H_2_0_2_ to initiate the reaction (final concentration 0.02%). Sections were then mounted and air dried before being dehydrated in an ascending ethanol series, cleared with xylene and coverslipped with Entellan (EM Science, USA).

For double-immunostaining, following the first reaction, sections were incubated in biotinylated goat-anti rabbit IgG (1:200) for 1 h. They were then incubated with Vectastain Elite ABC-HRP Kit for 1 h prior to incubation with Vector SG kit for chromogen staining (Vector Labs, USA). The sections were then mounted, air dried, dehydrated and cleared as described.

For immunofluorescence, sections were incubated with 50% ethanol and blocked with 10% NGS for 1 h, before overnight incubation with primary antibody (Supplementary Table 1). The following day sections were incubated with the relevant secondary antibodies for 2 h (1:400). Lastly, sections were stained with 0.3% Sudan Black in 70% ethanol to reduce background autofluorescence. They were then incubated with DAPI, mounted and coverslipped in PolyAquamount (Polysciences, Inc., USA).

### 2.5 Modified Bielschowsky stain

Coronal brain sections of control and R962-hTau rats were cut (8 μm), mounted alternating on gelatin-coated slides and dried overnight. The sections were stained using the modified Bielschowsky stain kit and protocol provided by American MasterTech (USA). Sections were dehydrated with ascending ethanol concentrations, cleared with xylene and coverslipped with Entellan.

### 2.6 Luxol fast blue

Control and transgenic tissue sections were mounted alternating on gelatin-coated slides, dried, then hydrated to 95% ethanol, and incubated in 1% Luxol fast blue solution (Sigma-Aldrich, Germany) for 8 h. The slides were then dehydrated in 95% and 100% ethanol, cleared with xylene and coverslipped with Entellan.

### 2.7 Microscopy and quantification

For brightfield microscopy, Axio Imager M2 microscope equipped with an AxioCam 506 color digital camera and the ZEN Imaging software (v2.3 Blue; Zeiss, Germany) was used. For fluorescence microscopy, image acquisition was performed using a LSM800 Confocal Microscope AxioObserver (Zeiss, Germany) and the ZEN Imaging software (v2.3, Black; Zeiss, Germany). Three μm *Z*-stacks were then merged to form maximum intensity projections. Image analysis was completed using ImageJ software (NIH, USA). To quantify the number of neurons and microglia, ImageJ was used to manually count the number of NeuN- or Iba1-immunoreactive cells per field of view. Three sections per animal and three images per section were used to generate a mean value for each region. For fluorescent signal quantification of GFAP immunoreactivity, percent area and total fluorescence were measured using ImageJ across two sections per animal in each region. The 10- and 18–20-month-old cohorts were processed and imaged independently, therefore quantification should not be compared between time points. Total fluorescence of HT7 and T49 confocal images were quantified with ImageJ.

Quantification of lateral ventricle, hippocampal and brain cross-sectional volumes was done on NeuN-stained sections, and completed as described (Shi et al., 2017). Using ImageJ, sections were isolated from the background using a thresholding algorithm to generate binary images for the morphometric analysis. Six coronal, single-hemisphere sections per animal, 320 μm apart were analysed. Volume was calculated using the formula: volume = (sum of area) × 0.32 mm. Volumetric analysis started at the rostral dorsal hippocampus and concluded at the caudal end of the dorsal hippocampus, corresponding to the bregma coordinates −2.30 and −4.16 in the rat brain atlas (Paxinos and Watson, 2007). Hippocampal size was measured by manually tracing the region of three hippocampal sections with the same formula applied. Additionally, three sections per animal, at the level of the anterior lateral ventricles, between bregma coordinates 1.75 and 0.75, were also analysed to reconstruct volumes of the region (Paxinos and Watson, 2007).

### 2.8 Magnetic resonance imaging (MRI)

Control and transgenic rats were randomly selected to undergo MRI at 15 months of age. Rats were anaesthetised with isoflurane (4–5%) and maintained at 1.5–2.5% throughout the duration of the imaging procedure. MRI studies were performed using Bruker Biospec 70/30 preclinical MRI scanner with a rat head surface coil. The 3D anatomical images were obtained using a steady-state free precession balanced sequence [repetition time/echo time = 5.2 millisecond/2.6 millisecond, flip angle = 30 degree, resolution = 0.231 × 0.231 × 0.116 mm, field of view = 44.416 × 44.416 × 22.208 mm].

### 2.9 Sarkosyl extraction

Sarkosyl-insoluble tau was extracted from cortex and hippocampus of 10-, 15- and 18-month-old control and R962-hTau rats, as well as from end-stage transgenic mice over-expressing the shortest human tau isoform with the P301S mutation (Allen et al., 2002) and age-matched control animals. Briefly, tissue was homogenised in A68 buffer (10 mM Tris PH 7.4, 0.8 M NaCl, 1 mM EGTA, 5 mM EDTA, 10% sucrose, 1 mM PMSF) containing protease and phosphatase inhibitors (Roche, Switzerland). After centrifugation at 20,700 ×*g* for 20 min at 4 °C, the pellet was resuspended in half the initial volume of buffer, homogenised, and centrifuged again. Supernatants were combined, and sarkosyl (Sigma, Germany) added to a final concentration of 1%. After a 1 h incubation, the samples were ultracentrifuged at 100,000 ×*g* for 1 h at 4 °C and the supernatants discarded. The pellets were washed and resuspended in 50 mM Tris-HCl, pH 7.4 (150 μl/g starting material).

### 2.10 Western blot

Samples from whole cortex were homogenised with A68 buffer containing protease and phosphatase inhibitors, then centrifuged at 13,000 ×*g* for 30 min. Sarkosyl-insoluble samples (diluted 1:10) and whole cortex or hippocampal homogenates were run on Novex WedgeWell 4–20% Tris-Glycine (Thermofisher, USA) or 12.5% SDS-PAGE gels, then transferred onto a nitrocellulose membrane (Bio-Rad, USA). Following blocking in PBS containing 0.1% Tween (PBS-T) and 1% BSA, membranes were incubated overnight at 4 °C with primary antibody (Supplementary Table 1). After 3 × 10 min washes in PBS-T, they were incubated in the relevant HRP-conjugated secondary antibody (Supplementary Table 1) for 1 h at room temperature. After 3 × 10 min washes in PBS-T, membranes were developed using enhanced chemiluminescence (GE Healthcare, USA) and X-Ray film (Fujifilm, Japan) or a CCD imaging system (AI600; GE Healthcare, USA). For comparison of species-specific tau and total tau levels between control and transgenic rats, whole cortex or hippocampal homogenates were used, densitometry quantified with ImageJ and values normalised to GAPDH and relative to age-matched control for T49 and Tau5.

### 2.11 Electron microscopy

Following deep anaesthesia, rats were perfused with cold saline for 45 s, followed by 4% PFA, and either 0.5% glutaraldehyde (morphology, 21 months) or 0.1% glutaraldehyde (immunogold and morphology, 12 months) for 1 min, then dropwise for the following 30 min. The fixative was then changed to 4% PFA solution for another 30 min before the brain was dissected and post-fixed in 4% PFA overnight. The following day, sections processed as described (Ribeiro-da-Silva et al., 1993). Briefly, sections were cut to 50 μm using a Vibratome (1000 plus) and collected in PBS. For immuno-electron microscopy (immuno-EM), sections were cryoprotected in sucrose-ethylene glycol, followed by permeabilisation through freeze-thaw cycles using liquid nitrogen-cooled isopentane. Each section underwent 3 cycles of 10 s to snap freeze and was allowed to thaw between each cycle before being recovered and washed. Sections were incubated with 1% sodium borohydride for 30 min, washed and blocked with 0.5% BSA and 10% NGS. The sections were then incubated with AT8 antibody (1:100; Thermofisher) overnight at 4 °C. The following day, they were washed in 0.5% fish gelatin, 0.8% BSA in PBS (pH 7.4) and incubated overnight using nanogold anti-mouse conjugated to 1.4 nm diameter gold (1:200; Nanoprobes, USA) at 4 °C. Sections for morphological evaluation and immuno-EM were washed, incubated in 1% glutaraldehyde for 10 min, washed again, and those with primary and secondary incubations underwent silver intensification for 8 min in the dark (Nanoprobes, HQ Silver, USA). Sections were then washed with water, treated with 1% osmium tetroxide at 4 °C for 1 h, dehydrated with ascending ethanol, followed by propylene oxide, incubated in increasing Epon solutions. They were then flat embedded in Epon between a coverslip and acetate foil and cured in an oven for 24 h at 60 °C. Sections were then examined using a light microscope and re-embedded in Epon blocks that were trimmed to generate a 1 mm^2^ area of interest. Ultrathin sections were cut on an ultramicrotome (Ultracut III, Reichert-Jung (Leica), Germany), then mounted on single-slot copper grids coated with Formvar, and subsequently contrast stained with uranyl acetate and lead citrate. Sections were observed with an FEI Tecnai 12,120 kV TEM (Thermofisher Sci., USA) and imaged using XR80C CCD camera system (AMT, USA).

For analysis of isolated filaments by EM, sarkosyl-insoluble tau was added to carbon-coated copper 400 mesh grids (EM Science, USA). Grids were then blotted, blocked with 0.1% gelatin, incubated with primary antibodies, followed by 10 nm gold-conjugated secondary antibodies in 0.1% gelatin (Supplementary Table 1). The grids were washed with water and negatively stained with 2% uranyl acetate.

### 2.12 Behavioural experiments

Rats were handled for 2 days prior to commencing behavioural testing and received a minimum of 2 days between subsequent behavioural experiments. For a timeline of behavioural testing, see Supplementary Fig. 1. All tests were carried out during the light phase of the circadian cycle, and rats spent a minimum of 30 min in the testing room prior to commencing the experiments. Rats with pododermatitis were excluded from completing the fear conditioning, Morris water maze and beam walking tests.

#### 2.12.1 Open field

Rats were placed in the arena (80 × 80 cm) and allowed to explore for 3 min. Their movements were automatically recorded using the tracking software EthoVision v3.1.13 (Noldus, The Netherlands). Times spent in the centre and perimeter of the box, and those spent exploring the environment, were recorded.

#### 2.12.2 Novel object

Each animal was subject to three phases of testing: exploration, novel object location and novel object recognition. Each trial was recorded by the tracking software EthoVision, and time spent exploring each object was manually recorded, as described (Iulita et al., 2014). During the initial exploration phase, five objects that had previously been tested to preclude spontaneous bias were placed in the arena. Animals were allowed to explore the five objects for 2 min. Animals were considered to be exploring when showing active interest in an object, we defined this as when the muzzle was touching, or in close proximity to the object, however, times in which the animal was touching an object, but not exploring the object were excluded. Each phase of the test was separated by a 20 min interval. During novel object location, one of the original objects was displaced to a new location within the arena, animals were allowed to explore for 2 min. During the novel object recognition task one of the original objects was replaced with a new object, again, each animal was allowed to explore for 2 min and exploration of each object was manually recorded.

#### 2.12.3 Morris water maze

Morris water maze was carried out as described (Hall et al., 2018). The maze, a circular, 170 cm diameter pool was half-filled with room temperature water, maintained at 22 °C ± 1, and made opaque by non-toxic white paint. Extra-maze visual cues were placed distally on the surrounding walls. Four orientation points, north, south, east and west, divided the pool into theoretical quadrants. The escape platform (12 cm diameter) was submerged below the surface 2 cm and located in the north-east quadrant.

On day 0, each animal was initially habituated to the water for 30 s, without the presence of the platform or extra-maze cues. Following habituation, each cohort was trained to find the fixed-position, submerged platform. Training was conducted over 5 days with four trials per rat per day. Rats were released from a different orientation between trials, and in a different sequence each day. This paradigm was adopted to ensure the use of extra-maze cues in locating the platform. Once the rat reached the platform, it was left there for 20 s before being retrieved. If the rat failed to locate the platform within 2 min, it was guided to, then left on the platform for 20 s. Each rat had a 20 min inter-trial period. On day six, rats underwent two trials, the first a 60 s probe test, where the platform was removed from the arena; the second, with a visible platform to assess for lack of motivation or sensorimotor deficits. All data was recorded with EthoVision 3.1.13.

#### 2.12.4 Fear conditioning

Fear conditioning protocol was completed as described (Iulita et al., 2014). Rats were subjected to 4 testing phases: habituation, conditioning, context recall and cued recall. Fear conditioning was paired with a context and an auditory stimulus that were repeated in the contextual and cued recall respectively. Rats were tested in the chamber, with a wire-grid floor and base connected to a weight transducer system to enable tracking of movement (Panlab, Spain). To evaluate fear, immobility (defined as freezing >2 s) was recorded by the software Freezing v1.3.01 (Panlab, Spain) during all phases of the test.

From day 1–3 the chamber was cleaned with 70% ethanol and scented with coconut extract between each animal. During habituation on day 1, animals were each placed in the chamber and allowed to explore for 300 s before being returned to their home cage. Day 2, rats underwent conditioning; after a baseline of 90 s of exploration, followed by the auditory conditioning stimulus, a 30 s tone (75 dB, 5 kHz) played and co-terminated with a 2 s foot shock (0.75 mA). Rats remained in the chamber for a further 120 s post-shock before being returned to their home cage. During day 3, contextual fear conditioning was assessed with rats placed in the chamber for 300 s. Cued recall was assessed on day 4. To create a different environment during the cued recall phase, visual cues were added to the chamber walls, wire-grid flooring replaced with a flat metal base, scented with peppermint extract and cleaned with 1% acetic acid between animals. Baseline exploration recorded for 120 s in the modified environment, followed by three consecutive tone presentations of 30 s (75 dB, 5 kHz), each separated by a 15 s pause.

#### 2.12.5 Beam walking test

The beam walking test consisted of three training sessions followed by three trials. All experiments were conducted on a beam 3 cm wide, 50 cm above a padded surface, with a dark goal box with bedding at the opposite end of the beam. Rats were trained to traverse the beam over three consecutive trials at increasing distances (25, 50 and 75 cm from the goal box) prior to commencing trials of 100 cm. Upon reaching the goal box, the rat was allowed to stay within for 20 s before commencing the next trial. Latency and footslips were recorded for each trial from the time the rat started to traverse. A failure to complete was allotted the maximum time of 20 s.

#### 2.12.6 Cognitive index

To assess the relationship between tau pathology and behaviour, we generated a global memory and learning score. This score, referred to as the cognitive index, was calculated based on the animals' performance across behavioural tests. Results from novel object location and recognition tasks, as well as fear conditioning percentages were converted to a 10-point scale. For Morris water maze, latency to platform zone during the probe trial was inversed and converted to a 10-point scale. Each rat score was averaged across tasks and expressed as a z-score.

### 2.13 Statistical analysis

Statistical analysis was completed using R 3.2.3 (R Core Team, Austria). Independent *t*-tests were performed, except when the F-statistic was significant, in which case Welch's t-test was applied. If the data violated underlying assumptions of parametric testing, Mann-Whitney *U* test was used. Where appropriate, multiple comparisons were made with t-tests and corrected with Bonferroni-Holm adjustments. Two-way ANOVA was used to analyse interactions and mixed design ANOVA was used to analyse data consisting of within and between subject factors. To assess the association between two variables, Pearson's correlation was used. All reported values are two-tailed, and overall significance was set at *p* < .05. Supplementary Table 2 details statistical output.

## 3 Results

### 3.1 Model generation and relative tau expression

The expression levels of rodent and human tau were compared by Western blotting using species-specific and total-tau antibodies (Supplementary Table 1). At 3 months of age, R962-hTau rats exhibited two-fold the total tau (Tau5) levels compared to age-matched WT littermates. Total tau hippocampal concentrations were approximately four times higher than age-matched controls between 6 and 15 month time points. Both total tau and human tau (HT7) levels subsequently declined at 18–20 months (Supplementary Fig. 2A). To exclude any effects on endogenous tau expression, we further investigated this at 3 months of age. We determined the increase in cortical tau levels in the R962-hTau rats was due to the expression of transgenic human tau (HT7), since no change in the expression of endogenous rodent tau (T49) was observed (Supplementary Fig. 2B).

### 3.2 Pathological changes in human and endogenous tau occur with age

**Fig. 1:**
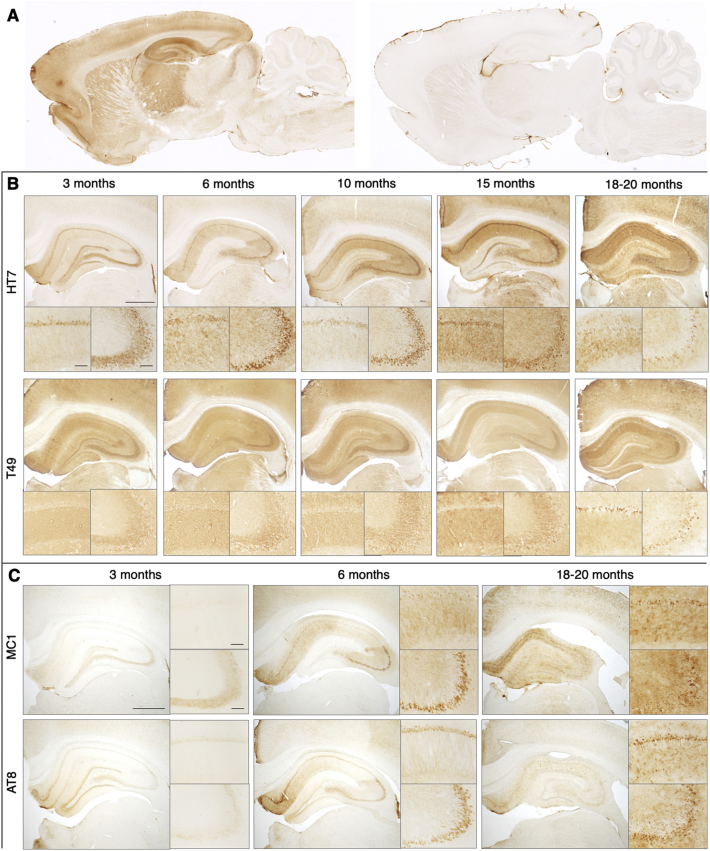
Hippocampal progression of tau pathology. Spatiotemporal progression of tau pathology in R962-hTau rats. (A) At the 10-month time point, sagittal brain sections show human tau (HT7) immunoreactivity throughout brain regions including cortical, hippocampal, thalamus and striatum (left), with immunoreactivity absent in control animals (right). Similarly, coronal sections of the hippocampus illustrate age-dependent increase in tau-reactivity. (B) Over the course of the pathology, human tau immunoreactivity transitions from being localized in the CA1 cell somas (lower left) and Mossy fibres (lower right) to the apical dendrites of the CA1 with a loss of Mossy fiber reactivity observed from 6-months. At later stages endogenous tau was also recruited to the cell soma. Another feature of note is the transition to more diffuse tau immunoreactivity. This was also observed using (B) total human tau HT7, (C) conformation dependent MC1 and phosphorylation dependent AT8. Insets show higher magnification images for the CA1 (top) and CA3 (bottom). *n* = 3–4 rats/group (3, 6, 15 months); *n* = 8–9 rats/group (10 and 18–20 months). Scale bar (B—C), 1000 μm; insets scale bar, 100 μm.

We investigated the extent and distribution of tau neuropathology present in the R962-hTau rat model between 3 and 20 months of age (Fig. 1). Using antibodies directed against human tau, endogenous rodent tau, and different tau posttranslational modifications, we observed age-dependent alterations in the tau immunoreactivity, reminiscent of the human tauopathies. As predicted by promoter activity and expression (Huang et al., 2012; Wang et al., 2013), HT7 immunoreactivity was present throughout the forebrain, but absent in control rats (Fig. 1A). An age-dependent increase in human-specific tau immunoreactivity (HT7) was observed to be strongest initially in the hippocampus (Fig. 1B) as well as the somatosensory, entorhinal and piriform cortices (Supplementary Fig. 3). Immunoreactivity was most prominent within cell somas and axons at 3–6-months of age, and appeared more dendritic in nature at later time points, localizing to the neuropil (Fig. 1B). Some immunoreactivity was observed in spinal cord and inferior colliculus, two regions where the CaMKIIα promoter is reportedly not expressed; however, immunoreactivity there was minimal, and no NFT-like inclusions were observed.

Rat tau (T49) became involved in the pathology only relatively late; endogenous aggregated tau was occasionally observed at 6- and 10-months. However, the consistent sequestration of rat tau was not observed until 18–20-months (Supplementary Fig. 4). Rat tau was particularly abundant in inclusions in layers II-III and V of the somatosensory cortex and in hippocampal neurons (Fig. 1B).

At early pathology stages, hyperphosphorylated and conformationally-altered tau immunoreactivity was observed throughout hippocampal formation and cortical regions (Fig. 1C). Phosphorylation-dependent immunoreactivity was largely localized to the fascioloa cinerea, subiculum and granular cell layer of the dentate gyrus, in addition to some cortical regions (somatosensory, entorhinal and piriform cortices) (Supplementary Fig. 5). At this stage, CA3 mossy fibres were immunopositive for MC1, however, mossy fibres at subsequent ages revealed no staining with MC1 or AT8 antibodies (Fig. 1C). This region continued to display T49-immunoreactivity in the majority of animals, indicating that endogenous tau was retained in these fibres (Fig. 1B). MC1-immunoreactivity also increased in other brain regions with age (Supplementary Fig. 6).

**Fig. 2:**
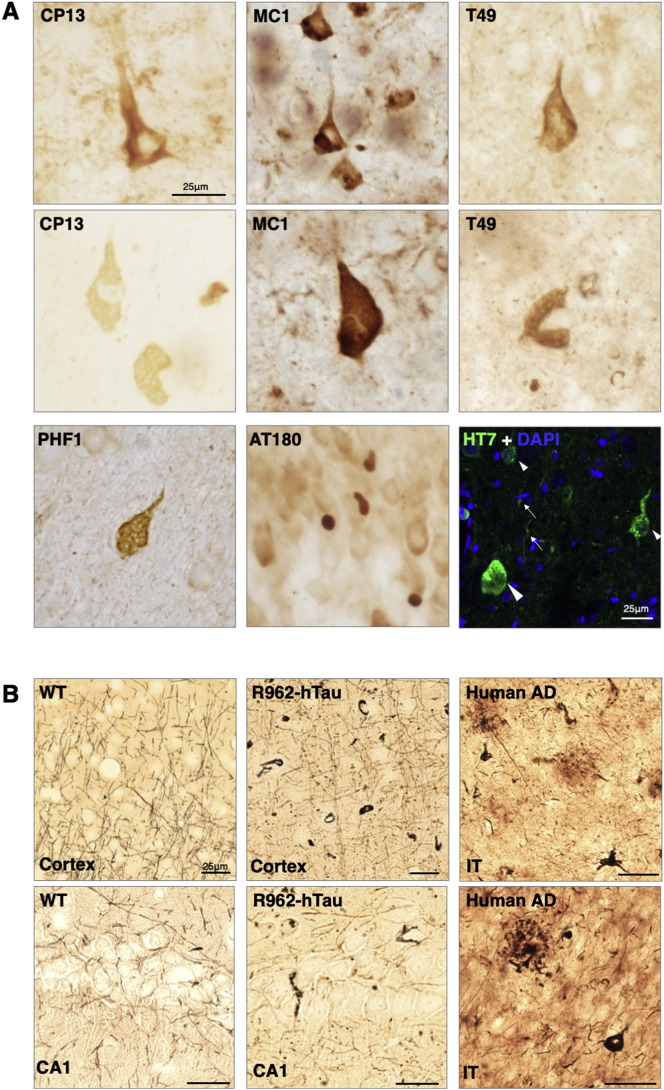
Neuronal tau pathology in R962-hTau rats. Tau pathology between 10 and 20-months of age (*n* = 8–9/group). (A) Early tau pathology may be seen at 10-months with phosphorylation-dependent antibody CP13 (top left) and conformational-dependent antibody MC1 (top centre). At the 18–20-month time point, there is extensive recruitment of endogenous tau, observed with T49-immunoreactivity, to tau inclusions throughout the cortex (top right). Later time points revealed ballooning neurons in the pyramidal cells of the cortex, immunoreactive for CP13, MC1, T49 and PHF1 (middle panels; bottom left panel). Some animals also develop AT180-immunoreactivity globose NFT that occur in the upper horn of the dentate gyrus granular cell layer (bottom centre). Immunofluorescence with HT7 (green) demonstrate the presence of tau burdened neurons (small arrowheads), dystrophic neurites (arrows) and an extraneuronal tangle, where no nuclear signal is observed with DAPI (bottom right). (B) Silver impregnation using modified Bielschowsky method reveals numerous silver positive inclusions at 15-months old in the R962-hTau cortex and CA1 region of the hippocampus, whilst the control cortex and CA1 are devoid of silver aggregates. Right hand panels demonstrate axons, plaque neurites and tangles in tissue from the inferior temporal lobe (IT) of a subject with AD (*n* = 2/group). Scale bars 25 μm.

Strong tau immunoreactivity and NFT-like inclusions were observed across cortical layers II-III, V and VI by 10 months of age (Fig. 2A, top left and top centre panels). By 18–20 months, NFT-like inclusions were also immunopositive for T49 (Fig. 2, top right). Many layer V pyramidal neurons of the somatosensory and somatomotor areas displayed neuronal ballooning (Fig. 2A, middle panels). These structures displayed both phosphorylation- and conformation-specific tau immunoreactivities, as well as T49-immunoreactivity. At later stages, some animals developed globular tau bodies in the granular cell layer of the dentate gyrus and some ballooning neurons were HT7-positive and many no longer had a nucleus, suggesting that they may have been the remnants of dead neurons, akin to extraneuronal or “ghost” tangles (Fig. 2A, bottom right panel). Other pathologies were also observed, including globose tau inclusions in the cortex and subiculum, and axonal spheroids, predominately in cortical regions (Supplementary Fig. 7A).

Immunoreactivity with phosphorylation-dependent anti-tau antibodies appeared to precede that with MC1. However, we observed variability between immunoreactivity for different tau antibodies and cellular localisation of tau at the same age (Supplementary Fig. 7B), a factor that was not dependent on sex.

Numerous inclusions were identified by modified Bielschowsky silver, throughout the cortex and hippocampus of R962-hTau rats (Fig. 2B, left and middle right panels). Axonal spheroids were also stained (Supplementary Fig. 7A). In control rats, only nerve cell axons were silver-positive.

### 3.3 Biochemical analysis reveals age-dependent accumulation of endogenous and human insoluble tau

Using a panel of anti-tau antibodies, sarkosyl-insoluble fractions from 10- and 18–20-month-old R962-hTau rats were analysed by Western blot. To ascertain how this compares to transgenic mouse models of tauopathy, samples were run alongside those from end-stage mice expressing the 0N4R human Tau with the P301S mutation under the control of the murine Thy1 promoter (Allen et al., 2002).

**Fig. 3:**
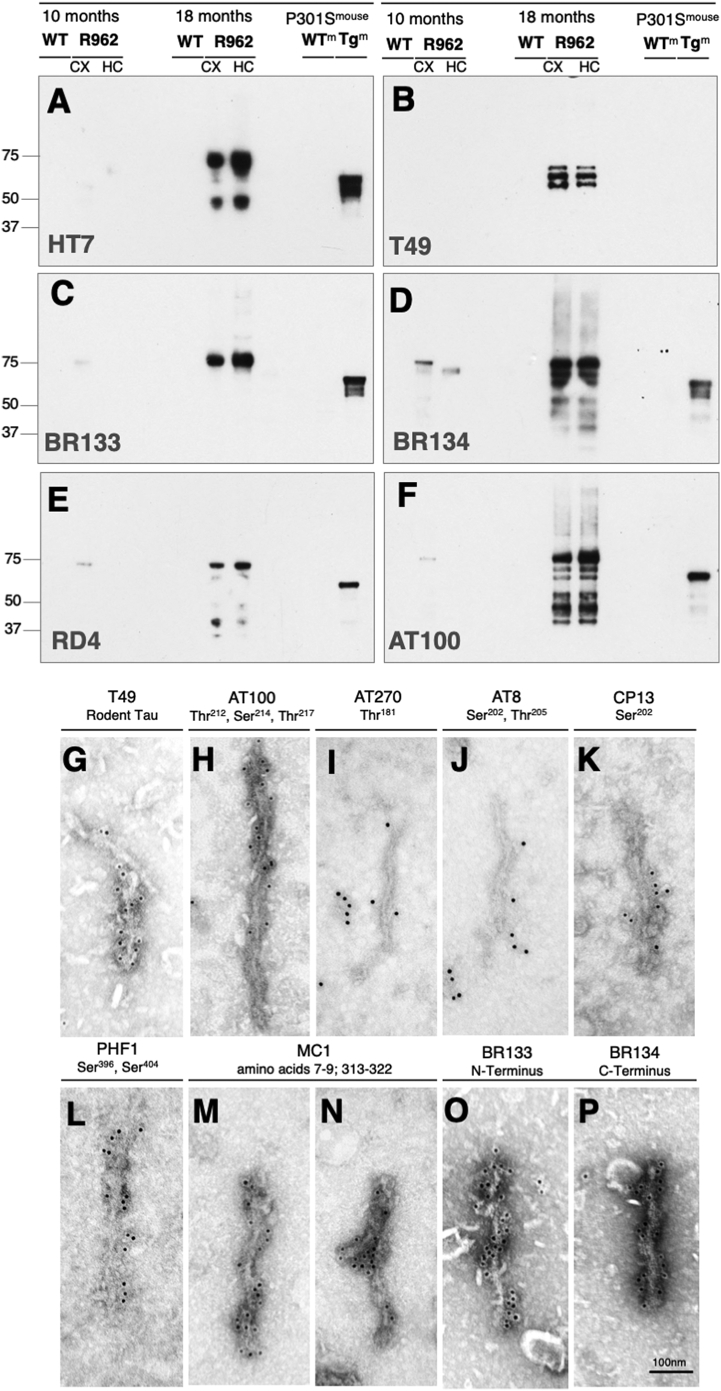
Analysis of sarkosyl-insoluble tau in the cortex and hippocampus. Western blotting of sarkosyl-insoluble tau at 10-months (left) and 18-months (centre) in R962-hTau cortex (CX) and hippocampus (HC) in comparison to the end-stage P301S mouse model of tauopathy, (right) (*n* = 4/group). (A) Both R962-hTau rats and P301S mice display human tau-immunoreactivity (HT7), (B) while only the R962-hTau rats have significant endogenous rat tau recruitment (T49). At 10-months of age, low levels of N-terminus (C; BR133) and C-terminus (D; BR134) immunoreactivity can be observed, with an increased abundance at the 18-month time point. (E) 4-repeat tau (RD4) is present at 10-months, and becomes more abundant in 18-month-old transgenic rats, (F) as well as the filament associated phosphorylation-dependent antibody, AT100. (G) Immunoelectron microscopy of sarkosyl-insoluble filaments of a 15-month-old R962-hTau rat confirmed endogenous tau recruitment using T49 (*n* = 1). (H-L) Presence of pathology-associated phosphorylation AT100, AT270, AT8, CP13 and PHF1. (M,N) We also observed MC1-immunoreactivity detecting conformational changes. (O, P) Similar to Western blotting, filaments were positive for BR133 and BR134. Scale bar (G–P) 100 nm.

Sarkosyl-insoluble fractions from control rats and mice did not exhibit any insoluble tau material. At 10 months of age, human aggregated tau was detected in cortex and, to a lesser extent, hippocampus of R962-hTau animals (Supplementary Fig. 8A), similar to the aggregation patterns observed by immunohistochemistry. This tau ‘load’ was greater in the old R962-hTau rats and comparable to that seen in P301S mice (Fig. 3A). Unlike the P301S mouse model where rodent tau was undetected, endogenous rat tau was present in the sarkosyl-insoluble fractions of 18–20-month-old R962-hTau animals (Fig. 3B).

In younger R962-hTau rats, insoluble tau was human (HT7 immunoreactivity) and ran predominantly as a band of around 75 kDa with no detectable rat tau (Supplementary Fig. 8A). At later time points, HT7-immunoreactivity appeared as bands of 45–75 kDa (Fig. 3A), and rat tau was recruited to insoluble forms that appeared as a triplet of 55–72 kDa (Fig. 3B).

Phosphorylation-independent anti-tau antibodies BR133 and BR134 were used to ascertain the presence of full-length tau (Fig. 3C, D). Lower molecular weight bands were observed with antibodies directed against the C-terminus, but not the N-terminus. No 3R tau isoforms (RD3) were detected in the sarkosyl-insoluble preparations, but 4R tau isoforms (RD4) were present (Fig. 3E). Immunoreactivity with AT100, an antibody that detects filamentous tau, demonstrated the presence of abundant tau filaments in the hippocampus and cortex at 18–20-months of age (Fig. 3F). Relatively low levels of AT100 were observed at the 10-month time point in cerebral cortex, but not hippocampus.

By negative-stain electron microscopy, sarkosyl-insoluble filaments from 15-month-old R962-hTau rats appeared as twisted ribbons. Sequestration of rat tau was confirmed by decoration with antibody T49 (Fig. 3G). Filaments were also immunoreactive with phosphorylation-dependent antibodies CP13 and PHF1 (Fig. 3H, K, L), as well as with conformational-dependent tau antibody MC1 (Fig. 3M, N), while only little immunoreactivity was observed with AT8 and AT270 (Fig. 3I, J). Full-length tau was present, as evidenced by filament decoration with BR133 and BR134 (Fig. 3O, P).

### 3.4 Neurodegeneration, neuronal cell loss and ventricular dilation is present in aged R962-hTau rats

**Fig. 4:**
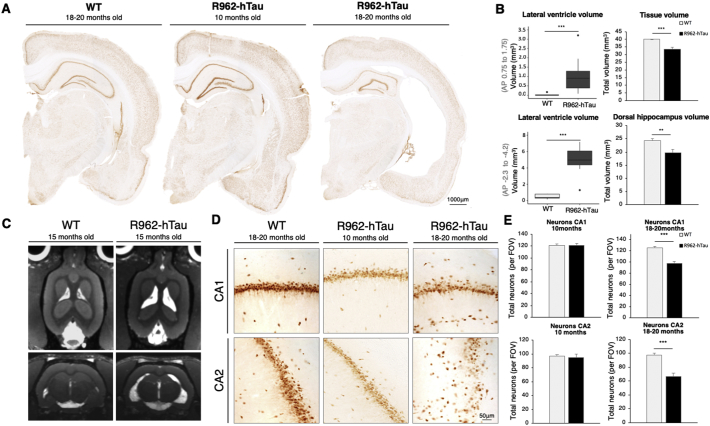
Ventricular dilation, hippocampal atrophy and neuronal loss in R962-hTau rats. (A) Coronal brain sections of an 18-month-old control, a 10-month-old R962-hTau and an 18-month-old R962-hTau rat demonstrating the extensive tau pathology-dependent ventricular dilation and hippocampal atrophy in the R962-hTau rats by 18-months of age. (B) Volumetric analysis of the lateral ventricles, forebrain and dorsal hippocampus of 18-months old rats (*n* = 8/group) show that ventricular volumes increase (left panels) while forebrain (top right panel) and hippocampal volumes decrease in R962-hTau rats (bottom right panel). AP coordinates 1.75 to 0.75 (forebrain); −2.30 to −4.20 (dorsal hippocampus)(Paxinos and Watson, 2007). (C) Representative images of transverse and coronal maximum intensity projections from T2-weighted MRI studies in 15-month-old animals, demonstrating ventricular dilation confirming histological observations and demonstrating these features are initiated by 15-months of age (*n* = 1/group). (D) Representative images of CA1 (top panel) and CA2 (bottom panel) of 18-month-old control, 10-month-old R962-hTau and 18-month-old R962-hTau rats, illustrating the marked neuronal loss and disorganization of the hippocampus. (E) Neuron counts based on average NeuN-immunoreactive nuclei per field of view (FOV) were generated from 3 images for each section for CA1 (top) and CA2 (bottom) (*n* = 7–8/group). Values are expressed as means ± SEM. Independent *t*-tests and Mann Whitney *U* tests were used where appropriate. **p < .01, ***p < .001 Scale bar: A, 1000 μm; D, 50 μm.

In order to characterise alterations in brain volume and structure, we performed morphometric analysis of coronal brain sections (Fig. 4A, Supplementary Fig. 8B). By 18–20-months of age, we found an increase in ventricle size (*p* = .001), a decrease in brain volume in the frontal cortex (*p* = .001) and hippocampus (*p* = .0057) (Fig. 4B). Brain atrophy and ventricular dilation were not observed in R962-hTau rats at the 10-month time point, despite progressive tau pathology (Fig. 4A). No interaction between sex and genotype for ventricle volume was observed [Genotype:Sex interaction, *p* = .48, not significant (NS)]. To confirm the histological observations of ventricular dilation and brain atrophy, we performed an MRI analysis of an individual rat at 15 months of age (Fig. 4C), which confirmed *in vivo* these prominent anatomical alterations akin to overall consequences of tauopathy in the human.

Given the alterations in brain structure, we counted the number of NeuN-immunoreactive nuclei in the hippocampus (Fig. 4D, E). No differences were observed in the 10-month-old cohort in layers CA1 and CA2. However, in transgenic rats aged 18–20-months, we observed an extensive loss of neurons throughout CA1 (*p* < .0001) and CA2 (*p* < .0001) (Fig. 4E).

### 3.5 Activation of microglia and astrocytes in regions with tau pathology

We investigated whether alterations in glial cell numbers and morphology occurred in R962-hTau rats. We used antibodies against microglial/macrophage ionized calcium-binding adapter molecule 1 (Iba1), and the astrocyte-specific protein, glial fibrillary acidic protein (GFAP).

**Fig. 5:**
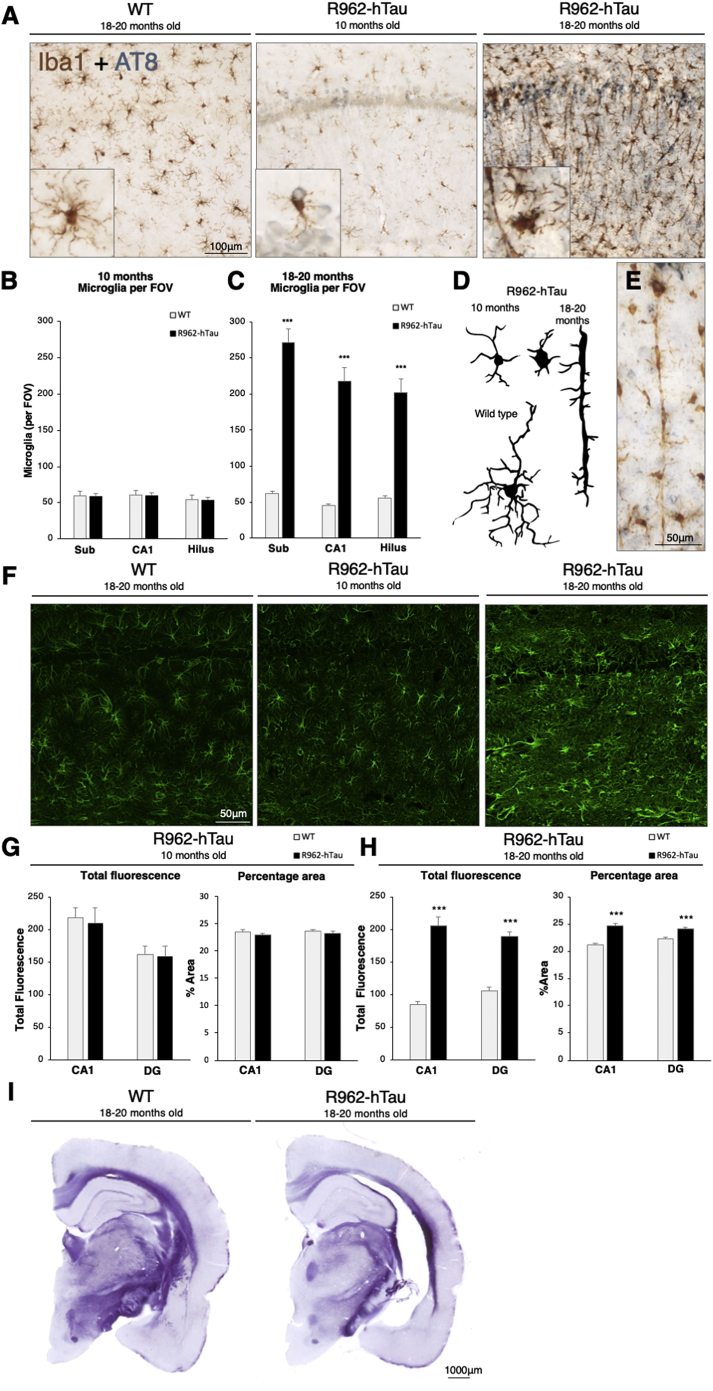
Microglia proliferation, rod-like microglia, astrocyte activation and myelin degeneration. (A) Representative images of Iba1-immunoreactivity in the CA1 region of the hippocampus, at 10- and 18–20-month-old rats, demonstrating both microglia proliferation and morphological changes occur by the late time point. Quantification of microglia numbers per field of view (FOV) in hippocampal regions subiculum (Sub), CA1 and hilus at the (B) 10 and (C) 18–20-month time points highlight the increase in microglia numbers (*n* = 7–8/group). (D) Microglia phenotypes at the two time-points are summarised. (E) Rod-like microglia form trains that run parallel to neuronal projections throughout hippocampal regions and the somatosensory cortex. (F) Representative images of hippocampal astrocyte GFAP-immunoreactivity demonstrating a more reactive phenotype in R962-hTau rats at the 18–20-month time point. This is supported by the quantification of the percent area covered, and fluorescence intensity in the CA1 and molecular dentate gyrus (DG) layers at the (G) 10- and (H) 18-month time points (*n* = 7–8/group). (I) Micrographs demonstrating apparent myelin loss in the R962-hTau 18-month-old rat compared to control rats (*n* = 6/group). Scale bar, A 100 μm; E,F 50 μm; I 1000 μm. Values are expressed as means ± SEM. Bonferroni-Holm corrected Welch's *t*-tests were used. ***p < .001.

At 10 months of age, the number of microglia in R962-hTau rats was comparable to that seen in control animals. In contrast, dramatic increases in the number of microglia in the subiculum (*p* = .0002), CA1 (Fig. 5A; *p* < .0001) and the hilus of the dentate gyrus (*p* < .0001) were observed in 18–20-month-old transgenic rats (Fig. 5C).

Some microglial cells were seen to localise around AT8-immunopositive neurons (Fig. 5A, centre panel inset), however, there were no differences in microglial numbers at 10 months of age between transgenic and controls rats (Fig. 5B). At later time points (18–20-months), along with extensive proliferation (Fig. 5C), microglia displayed an activated phenotype (Fig. 5D). Additionally, some microglia adopted a distinct, rod-like phenotype, which has previously been described in AD patients and in experimental models of traumatic brain injury (Bachstetter et al., 2015). Rod-like microglia ran parallel, either individually or in trains (Fig. 5E), to neuronal projections from the fascioloa cinerea, subiculum, CA1 and layers III and V of the primary somatosensory and entorhinal cortices respectively.

Alterations in astrocytic GFAP expression were also quantified at 10 and at 18–20 months of age. No differences were observed at 10 months when compared with age-matched controls (Fig. 5F, G). However, increases in GFAP total fluorescence and fluorescence area was observed in CA1 and the molecular layer of the dentate gyrus at 18–20-months (Fig. 5F, H).

### 3.6 Extensive myelin degeneration occurs in aged R962-hTau rats

To determine if a loss of white matter was associated with the degeneration in grey matter, we stained tissue sections with Luxol Fast Blue. This revealed demyelination in the corpus callosum of transgenic rats when compared with control animals at 18–20-months, indicating this pathology occurs secondary to upstream tau aggregation (Fig. 5I).

### 3.7 R962-hTau rats develop marked glial and neuronal ultrastructural pathology

**Fig. 6:**
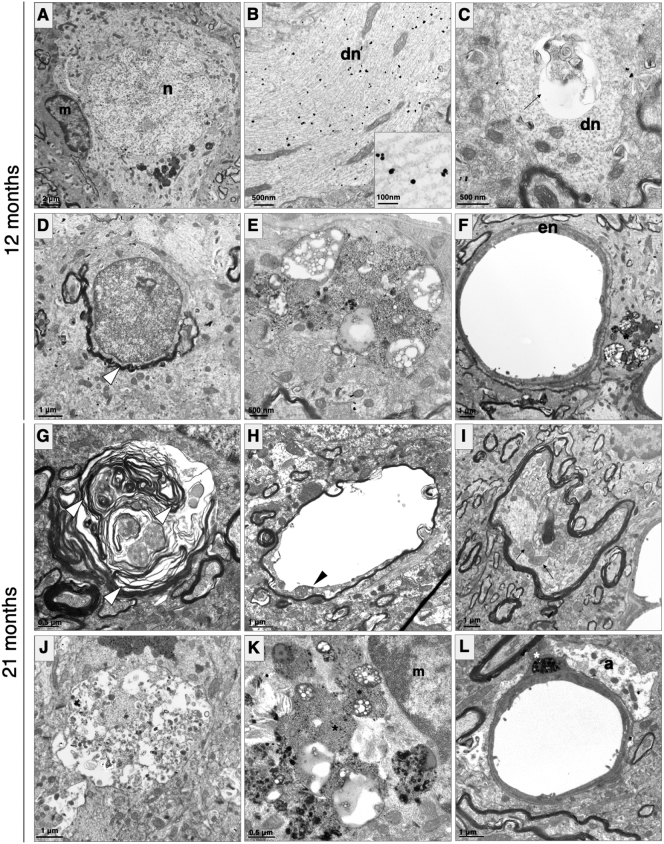
Ultrastructural morphology. Representative transmission EM images from 12- and 21-month-old R962-hTau rats revealing numerous ultrastructural degenerating pathological features (*n* = 1–2/group). (A) At the 12-month time point, microglia (*m*) are observed localizing adjacent to neuronal cell bodies (*n*) with numerous lysosomal bodies and AT8 immunogold reactivity within the soma. (B) AT8 tau-immunoreactivity is present throughout dendrites (*dn*) of these neurons. (C) In addition to tau-immunoreactivity, dendrites display vacuoles. Other abnormalities are present, including the occurrence of redundant myelin (D, arrowhead). (*E*-F) Amorphous debris are abundant at the 12-month time point, including lipid droplets and debris found adjacent to and within vascular components and endothelial (*en*) blebbing. (G) The later time point revealed extensive axonal abnormalities including myelin degeneration and the formation of concentric membranous whorls, with a distinct loss of compact myelin. (H) Axonal myelin balloons, whereby an axon (arrowhead) was internally displaced and pushed up against one side of the myelin with extensive thinning of the myelin and sheath splitting. (I) Extremely swollen mitochondria (arrows) occur within an irregular myelin sheath. (J) A possible axonal spheroid with autophagic vacuoles, myeloid bodies and filaments. (K) Microglia (*m*) with internalised electron-dense and electron-lucent debris (asterisks). (L) Vascular abnormalities are present, including pericyte debris (asterisks) and swollen astrocytic end feet.

We used transmission EM to investigate ultrastructural changes. At 12 months of age, microglia were often observed to localise alongside neurons positive for tau by immunogold labelling (Fig. 6A), thus confirming our findings from light microscopy at 10-months (Fig. 5A, centre panel inset). In addition to the cell soma, AT8 immunoreactivity was also found in association with microtubules in axons and dendrites (Fig. 6B). Instances of axonal and dendritic abnormalities, such as formation of vacuoles (Fig. 6C), as well as redundant myelin (Fig. 6D), were observed, indicating that axonopathy was initiated prior to this time point. Additionally, amorphous debris were particularly abundant alongside blood vessels (Fig. 6E) and were present in vascular components, such as pericytes (Fig. 6F). Endothelial blebbing was also observed (Fig. 6F).

Investigation at a later time point (21-months) revealed extensive myelin breakdown, forming concentric multi-lamellar myelin whorls, and a distinct loss of compact myelin (Fig. 6G). Additionally, some myelinated axons were observed to balloon, a phenomenon caused by the splitting of the intraperiod line of the myelin sheath and the formation of a fluid-filled vacuole, resulting in lateral axonal compression (Feldman and Peters, 1998) (Fig. 6H, arrowhead). Other cellular abnormalities included axonal spheroids and degenerating regions containing filaments, autophagic vacuoles and myeloid bodies (Fig. 6I, J). Microglia that appeared to have phagocytosed electron-dense and lipid-based amorphous debris were also observed (Fig. 6K). Vasculopathy became more apparent at this later time point, with swollen astrocytic end-feet and debris accumulating within vascular components, including pericytes (Fig. 6L).

### 3.8 R962-hTau rats develop severe impairments in memory and learning at late tau pathology stages

#### 3.8.1 Novel object

**Fig. 7:**
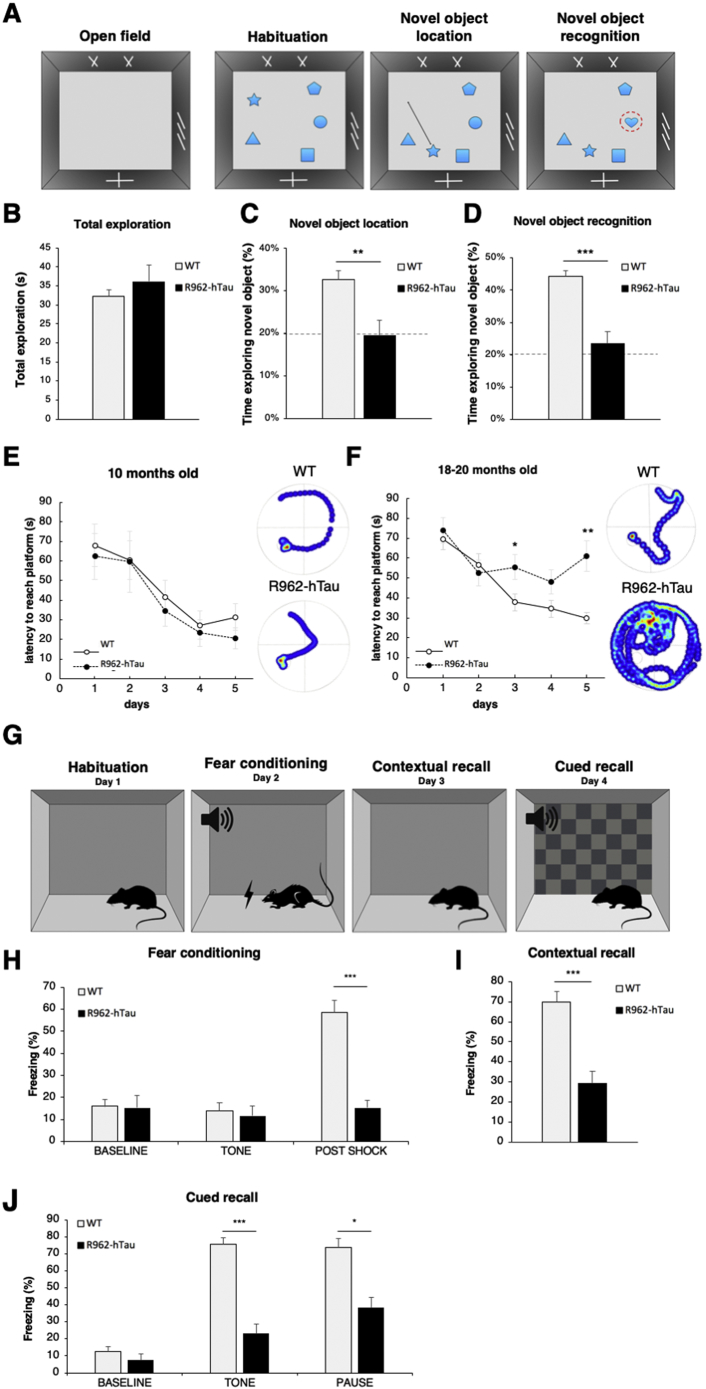
Behavioural studies reveal numerous memory and learning deficits. Behavioural studies were conducted at 10- and 18–20 months of age (A) The open field and novel object location and recognition paradigms. (B) At 18–20 months of age no differences between genotypes were observed in the open field task. However, R962-hTau rats showed extreme deficits in (C) novel object location and in (D) novel object recognition tasks. (E) In the learning phase of the Morris water maze task, no differences in the latency to reach platform were identified in 10-month-old rats, where both transgenic and control animals learnt the task. Representative search patterns of the final training day (day 5) are depicted on the right. (F) The 18–20-month cohort revealed R962-hTau rats had attenuated learning curve, and did not consistently improve, whereas performance of the control rats improved over time. (G) Fear conditioning was adopted to assess cued and contextual recall. (H) Fear conditioning at 18–20 months of age demonstrates R962-hTau rats have a response comparable to base-line after receiving the foot shock and subsequently fail to freeze comparably to control animals in the (I) contextual recall phase or (J) cued recall tone and pause phases. Values are expressed as means ± SEM (*n* = 14–16 WT; 8–9 R962-hTau/group). Welch's and independent *t*-tests were used dependent on the outcome of F-tests for variance and followed by Bonferroni-Holm correction. Mixed model ANOVA was used to analyse Morris water maze across days *p < .05, **p < .01, ***p < .001.

To evaluate different paradigms of memory and learning, a battery of behavioural tests were completed at 10- and at 18–20-months. To assess working memory, we employed novel object testing (Fig. 7A). At 10 months, R962-hTau rats performed comparably to controls in all testing phases (Supplementary Fig. 9A–D). At 18–20 months, there were no differences in overall exploration during the initial phase, indicating no spontaneous bias for an object (Fig. 7B). During testing phases, however, relative to control rats, R962-hTau rats were unable to recognize the object during the re-location task (Fig. 7C; *p* = .008) and, similarly, did not spend extra time with the novel object (Fig. 7D; *p* = .0002).

#### 3.8.2 Morris water maze

To test visual-spatial memory, we employed the Morris water maze test. While 10-month-old transgenic rats performed similarly to controls (Fig. 7E), at 18–20 months of age, there was a significant Genotype:Training day interaction (*p* = .034) and significant main effects for both genotype (*p* = .032) and training day (*p* < .0001). The failure of R962-hTau to learn the task by day 5 supports this (Day 5; *p* = .008). Representative images of searching patterns during day 5 illustrates directed-searching for both genotypes at 10-months of age, and in 18–20-month-old controls, but random-searching in 18–20-month-old R962-hTau rats (Fig. 7E, F). These results are consistent with severe impairments in memory and learning. During the probe trial, the platform was removed, and latency to reach the platform zone and entries into the platform zone recorded. The probe trial followed a similar trend with a tendency for WT rats to have lower latency times to the platform area and higher number of entries into the platform zone, however, these failed to reach significance (Supplementary Figs. 9F and 10A, B). This is probably due to insufficient power or the intrinsic phenotypic variations. A factor perhaps not as relevant across training days due to training repeats. During the visible platform trial, all rats reached the platform, thus no rats were excluded on the basis of sensorimotor deficits or lack of motivation.

#### 3.8.3 Fear conditioning

We studied fear conditioning to evaluate cued and contextual memory (Fig. 7G). This paradigm enables testing of associative memory where a conditional stimulus (foot-shock) is paired with an unconditional stimulus (tone). During contextual recall, the rats are placed in the same context after 24 h but not submitted to any tone or foot-shock. In the 10-month-old cohort, both control and R962-hTau rats displayed similar response across testing phases (Supplementary Fig. 9G–J). At 18–20 months-old, control and transgenic rats had similar levels of freezing at baseline and initial tone; however, 18–20-month-old R962-hTau exhibited a severely attenuated freezing response immediately following the shock (*p* < .0001) (Fig. 7H). During the contextual recall, old R962-hTau rats froze significantly less than controls (*p* < .0001) (Fig. 7I). Similarly, in the cued recall condition, freezing was greatly reduced in transgenic animals compared to controls, including during both tone (*p* < .0001) and pause conditions (*p* = .009); however, there was no difference in baseline freezing [*p* = .412 (NS)] (Fig. 7J).

#### 3.8.4 No deficits in motor coordination

The beam walking test demonstrated that no apparent motor deficits in prehensile grip strength or motor coordination were present in the 18–20-month-old rats, as assessed through latency to cross the beam and number of footslips (Supplementary Fig. 10E–G). This was paralleled by no differences in swimming velocity (Supplementary Figs. 9E and 10C). Since hyperactivity has been reported to occur in other tauopathy models, we completed the open field task to assess total exploration, and assessed overall freezing at baseline in fear conditioning, which demonstrated no significant difference between genotypes (Supplementary Figs. 9A, G and 10D).

### 3.9 Human tau levels correlate with neurodegeneration, but not cognitive index

To assess if behavioural deficits correlated with human tau levels, we compared the immunofluorescence HT7 signals across regions of interest (CA1, CA3, hilus, amygdala cortex and basomedial nuclei, somatosensory cortex) with each rats' cognitive index score, representing a composite of their performance across all cognitive tests, and brain volume in the same region (Supplementary Fig. 11). While human tau levels did not correlate with cognitive indices [Supplementary Fig. 11B; *r* = 0.16, *p* = .70 (NS)], they did correlate with brain volume in the same region (Supplementary Fig. 11C; *r* = 0.82, *p* = .012). At 18–20 months of age animals with less intense HT7-immunoreactivity generally had greater brain atrophy and less intense inclusions (Supplementary Figs. 3 and 11A). The same association was present for levels of endogenous tau (T49) across the regions of interest (Supplementary Fig. 11D). T49 levels in R962-hTau rats did not correlate with cognitive index [Supplementary Fig. 11E; *r* = −0.001, *p* = .99 (NS)], but positively correlated with brain volume (Supplementary Fig. 11F; *r* = 0.77, *p* = .027). These results likely reflect a reduction of brain tau levels following neuronal loss.

## 4 Discussion

In this report, we describe the production and characterisation of the first rat line transgenic for full-length human tau with the P301S mutation. We demonstrate the progressive, age-dependent increase in the accumulation of filamentous tau, which was upstream of neurodegeneration, myelin abnormalities, gliosis and behavioural deficits. In contrast to the transgenic mouse model where human P301S tau forms filamentous aggregates (Allen et al., 2002), aggregates of the R962-hTau rats comprised both human transgenic tau as well as endogenous rat tau in older timepoints.

Rat models of tauopathy have been generated. Notably, the first report of transgenic rat lines expressing human truncated tau demonstrated the brain occurrence of sarkosyl extracted tau fibrillary material consisting of both endogenous and human tau (Filipcik et al., 2012; Zilka et al., 2006). Furthermore, these truncated models described the presence of inclusions within the spinal cord and brainstem, resulting in motor and coordination deficits early and independently of neuronal loss (Valachova et al., 2018). In these models, no neuron loss was detected in the hippocampus or cortex (Filipcik et al., 2012; Koson et al., 2008). A rat model of CNS tauopathy resulting from the transgenic expression of the full-length human tau with the FTDP-17T-associated P301L mutation, was reported by Korhonen and collaborators (Korhonen et al., 2011). This model, however, displayed minor tau pathology, and an absence of neuronal loss and behavioural impairment. This difference is likely attributable to a weak transgene expression; the authors reported around 30% of their transgenic rats failed to produce detectable levels of either human tau protein or mRNA even though they utilised a mouse prion protein (mPrP) promoter, which usually enables a high transgene expression (Korhonen et al., 2011). Furthermore, the mPrP promoter drives transgene expression to regions differing from those where the CamKIIα promoter is active, for example in the spinal cord (Maskri et al., 2004). The P301L and P301S are both missense mutations located within exon 10 and are associated with FTDP-17. These mutations both strongly reduce microtubule assembly, albeit a smaller effect is observed with P301S compared to P301L (Bugiani et al., 1999).

Tau transgenic mice have been more frequently used and recapitulate, to varying extents, important disease features. Among the most commonly used models are those with the P301S and P301L mutations, including 0N4R P301S, PS19 and Tg4510 models which develop hallmarks of tauopathy, including neuronal loss, gliosis and cognitive impairment (Allen et al., 2002; Ramsden et al., 2005; Yoshiyama et al., 2007). However, behavioural deficits occur relatively early in across these models, opposing the relatively late development of cognitive impairments in humans. Additionally, many fail to develop a marked, human-like, brain atrophy and significant ventricular dilation. In this regard, the PS19 mouse model achieved these features after the co-expression of P301S mutant tau and human ApoE3 and ApoE4 isoforms (Shi et al., 2017). The closer genetics and physiology of rats to humans may enable a more in-depth analysis of the key events marking the progression of tau pathology and how they relate to cognitive impairment and neurodegeneration. Evidence indicates that endogenous rat ApoE may be functionally more similar to human isoforms, and has a structural homology of 73.5% and 73.9% with ApoE3 and ApoE4 respectively (Tran et al., 2013). This may be significant for a more faithful recapitulation of human tauopathy in rat models, models which may be more suitable to experimental therapeutics with better possibilities of clinical translations.

Varying levels of tau pathology can be observed in cognitively normal individuals, complicating the faithful recapitulation in animal models of key stages occurring in human tauopathies (Braak and Braak, 1991; Braak and Del Tredici, 2011). In most tauopathies including AD, primary age related tauopathy and FTDP-17T, tau pathology is present to some extent decades before the manifestation of clinical symptoms (Braak and Braak, 1991; Spillantini et al., 2000). Analogously, pathology-associated tau modifications were present in R962-hTau transgenic rats, including tau hyperphosphorylation and conformational changes, as well as low levels of insoluble tau filaments, at a time when R962-hTau have intact cognitive functions. Thus, measurable memory dysfunction does not occur coincidentally with the earlier tau pathology in our rat model, as is the case with the early tau pathology observed in cognitively normal individuals. On the other hand, at later stages in the evolution of tauopathies, such as dominantly inherited AD, an elevation in cerebrospinal fluid tau coincides with drastic changes in cognitive scores during the transition to the clinical presentation of the disease (McDade et al., 2018). At very advanced stages of tau pathology we observed neuronal loss, ventricular dilation and myelin compromise leading to cognitive decline, as is observed in humans. The fact that the rat does not manifest cognitive impairment at the early stages of tau pathology, as opposed to mouse models, would indicate a higher level of neural reserve in the rat.

Myelin degeneration has been observed in patients with mild cognitive impairment and end-stage AD. Demyelination has also been observed in cortical regions and the corpus callosum (Bouhrara et al., 2018), and correlates with cognitive decline (Vermersch et al., 1996). Axonal myelin vacuolisation and degeneration have been described by EM of biopsies from AD brain (Terry et al., 1964). Comparable to these observations, our EM studies on the brains of the present transgenic rat model also revealed numerous axonal varicosities, myelin vacuolisation and ballooning, as well as signs of Wallerian degeneration. Forms of myelin degeneration have similarly been reported in other models to varying degrees, including minor myelin disruptions in a triple-transgenic mouse model of AD (Desai et al., 2009) as well as more progressive myelin pathologies in some transgenic tau mice expressing human 4R tau (Andorfer et al., 2005; Probst et al., 2000). Furthermore, glia specific models of tauopathy that develop pathology specifically in astrocytes or oligodendrocytes display signs of axonopathy and Wallerian degeneration in the spinal cord (Forman et al., 2005; Higuchi et al., 2005). These pathologies may be a consequence of axonal degeneration as well as glial cell dysfunction leading to myelin pathology. Additionally, neuronal expression of tau has also been shown alter oligodendrocyte progenitor cell proliferation and differentiation in the context of focal white matter demyelination (Ossola et al., 2016). While the contributions of myelin pathology to disease progression in this rat line remain enigmatic, it is clear that a relationship exists between it and cognition in aging and disease in humans (Bartzokis, 2004; Bartzokis, 2011).

In addition, we observed in this transgenic model extensive microglial cell proliferation and activation coincident with the onset of tau-related neurodegeneration. While microglial activation has been widely described to occur in parallel to neurodegeneration, less is known about rod-like microglia (Bachstetter et al., 2015). It has been suggested that they may be protective (Taylor et al., 2014), but their true function remains to be discovered. By EM, we observed many microglial cells with ingested debris, the bulk of which was amorphous and tau-negative. Whereas it has been suggested that microglial activation and inflammation can contribute to tauopathy (Leyns and Holtzman, 2017), a failure of the neurosupportive functions of astrocytes and cellular senescence may also play a role (Bussian et al., 2018; Sidoryk-Wegrzynowicz et al., 2017).

Ultrastructurally, the majority of tau filaments were twisted ribbons, similar to what has been described in FTDP-17T (Ingram and Spillantini, 2002). Decoration of these filaments confirmed pathology-associated modifications, findings which were mirrored by immunohistochemistry and Western blotting. The high-resolution structures of these filaments remain unknown.

How these pathological features impact cognitive function is essential for understanding disease progression and for assessing potential therapies. Paradigms, such as the novel object recognition task, rely on visual recognition memory, with hippocampal and parahippocampal structures associated with memory consolidation and retention. It is difficult to ascertain the precise CNS areas contributing to the impairments in acquisition and retention given the extensive pathology observed in the R962-hTau rats. However, given the short inter-trial period during the novel object testing, it appears likely that both hippocampal and parahippocampal areas were implicated, since R962-hTau rats failed to perform above chance levels. Similarly, the poor performance in the novel object location and Morris water maze tasks indicated a widespread failure in memory.

The virtual abolition of freezing after a shock and the failure to translate this to contextual and cued memory highlights alterations in fear processing in the amygdala and hippocampus of transgenic rats. Whereas contextual stimuli are processed in the hippocampus through connections with the basomedial and basolateral nuclei, impairments in contextual and cued memory indicate deficits at the level of the amygdala (Phillips and LeDoux, 1992). These cognitive deficits are only incipient well after tauopathy onset and concomitant with significant tau pathology, brain atrophy and nerve cell loss, recapitulating the sequence of events occurring in human tauopathies.

Among the lessons learned during the creation and characterisation of this model was that a relatively high expression of mutant tau is required to achieve the severe phenotype observed in the R962-hTau rats, as compared with the other rat models available. Choice of promotor also represents an important consideration, particularly when attempting to investigate cognitive changes. We are presently in the process of recreating a transgenic line with comparable expression, which will allow us and others to further investigate the intriguing relationship between advanced tau pathology, neuronal loss, ventricular dilation and myelin compromise.

In summary, this study supports the idea that rats transgenic for full-length human mutant tau are promising models, particularly, for studying the spatio-temporal progression of tau pathogenesis and how it relates to neurodegeneration. In this regard, this model has shown that tau aggregation lies upstream, followed by the sequestration of endogenous tau, microglia proliferation, reactive astrocytes, myelin abnormalities, neuronal loss, ventricular dilation, brain atrophy, concurrent with development of memory and learning deficits. It highlights that rat brains show resilience to the initial tau pathology and that marked cognitive impairments are coincidental with advanced tau pathology and neurodegeneration, a situation resembling the evolution and clinical presentation of AD and other tauopathies.

## Appendix A Supplementary data

Supplementary material Image 1
