# Supplementary material for: Neuropathological changes and cognitive deficits in rats transgenic for human mutant tau recapitulate human tauopathy
Source: Neurobiol Dis. 2019 Jul;127:323–38. doi: 10.1016/j.nbd.2019.03.018 (PMC6597947; doi:10.1016/j.nbd.2019.03.018)
Supplement: Supplementary file 1 — Supplementary material [file mmc1.pdf]

Supplementary Material

Neuropathological changes and cognitive deficits in rats transgenic for human mutant tau recapitulate human tauopathy

Janice C. Malcolm, Lionel Breuillaud, Sonia Do Carmo, Hélène Hall, Lindsay A. Welikovitch, Jennifer A. Macdonald, Michel Goedert, A. Claudio Cuello

Supplementary table 1. *List of primary and secondary antibodies used and their applications*

Antibody	Epitope/target	Supplier	App.	Conc.	Secondary Antibody	Conc.	Supplier
**Conformational Tau**							
MC1	aa 7-9; aa 312-322	Peter Davies	IHC	1:500	Goat anti-mouse IgG	1:200	MP biomedical
			EM	1:10	10nm gold-conjugated anti-mouse	1:50	BBI Solutions
**Phosphorylated Tau**							
AT100	$Thr^{212}/Ser^{214}/Thr^{217}$	Thermofisher	WB^S	1:1000	HRP conjugated goat anti-mouse	1:5000	Bio-Rad
			EM^S	1:50	10nm gold-conjugated anti-mouse	1:50	BBI Solutions
AT270	Thr^{181}	Thermofisher	EM^S	1:50	10nm gold-conjugated anti-rabbit	1:50	BBI Solutions
AT8	Ser^{202}/Thr^{205}	Thermofisher	IHC	1:500	Goat anti-mouse IgG	1:200	MP biomedical
			EM	1:100	1.4nm gold-conjugated anti-mouse	1:200	Nanogold
			EM^S	1:50	10nm gold-conjugated anti-mouse	1:50	BBI Solutions
AT180	Ser^{231}	Thermofisher	IHC	1:500	Goat anti-mouse IgG	1:200	MP biomedical
			EM^S	1:50	10nm gold-conjugated anti-mouse	1:50	BBI Solutions
CP13	Ser^{202}	Peter Davies	IHC	1:2000	Goat anti-mouse IgG	1:200	MP biomedical
			EM^S	1:50	10nm gold-conjugated anti-rabbit	1:50	BBI Solutions
PHF-1	$Ser^{396/404}$	Peter Davies	IHC	1:4000	Goat anti-mouse IgG	1:200	MP biomedical
			EM^S	1:50	10nm gold-conjugated anti-rabbit	1:50	BBI Solutions
**Tau Epitopes (phosphorylation independent)**							
BR133	N-terminus	Goedert lab	WB^S	1:4000	HRP-conjugated anti-rabbit IgG	1:4000	Bio-Rad
			EM^S	1:50	10nm gold-conjugated anti-rabbit	1:50	BBI Solutions
BR134	C-terminus	Goedert lab	WB^S	1:4000	HRP-conjugated anti-rabbit IgG	1:4000	Bio-Rad
			EM^S	1:50	10nm gold-conjugated anti-rabbit	1:50	BBI Solutions
RD3	3R tau	Merck Millipore	WB	1:2000	HRP-conjugated anti-mouse IgG	1:5000	Bio-Rad
RD4	R2, 4R tau	Merck Millipore	WB^S	1:2000	HRP-conjugated anti-mouse IgG	1:5000	Bio-Rad
**Rodent Tau**							
T49	Rodent tau	Merck Millipore	IHC	1:2500	Goat anti-mouse IgG	1:200	MP biomedical
			WB^S	1:50000	HRP-conjugated anti-mouse IgG	1:5000	Bio-Rad
			EM^S	1:50	10nm gold-conjugated anti-rabbit	1:50	BBI Solutions
			IF	1:4000	Alexa Fluor 568	1:400	Thermofisher
**Total Tau (human)**							
HT7	Human tau;	Thermofisher	IHC	1:1500	Goat anti-mouse IgG	1:200	MP biomedical
	aa 159-163		IF	1:750	Alexa Fluor 488/Alexa Fluor 568	1:400	Thermofisher
			WB^S	1:2000	HRP-conjugated anti-mouse IgG	1:5000	Bio-Rad
**Other**							
Iba1	Microglia/ Macrophages	Wako	IHC	1:2000	Biotinylated goat anti-rabbit	1:200	Vector labs
NeuN	Neuronal nuclei	Abcam	IHC	1:2000	Goat anti-mouse IgG	1:200	MP biomedical
GFAP	Astrocytes	Novus	IF	1:2000	Alexa Fluor 488	1:400	Thermofisher
DAPI	Nuclei	Sigma	IF	1:200000	N/A	N/A	N/A
GAPDH	GAPDH	Merck Millipore	WB	1:10000	HRP-conjugated anti-mouse IgG	1:5000	Bio-Rad

Amino acids (aa); Application (App.); Concentration (Conc.); Horse-radish peroxidase (HRP); Western blot (WB); Immunohistochemistry (IHC); Immunofluorescence (IF); Electron microscopy (EM); 3 repeat (3R); 4 repeat (4R); Repeat 1 (R1); Repeat 2 (R2); Repeat 3 (R3); Repeat 4(R4); ^{S} Sarkosyl preparations

Supplementary table 2. *Statistical output*

	Mean		SEM		p-value	Degrees of freedom	Test statistic	Statistical test used
	WT	R962	WT	R962				
**18-20 month-old cohort**								
Volumetric analysis								
Ventricle volume (AP-0.75 to -1.75)	0.03	1.10	0.02	0.36	0.0013	14	W = 1	Mann-Whitney
Ventricle volume (AP 2.3 to 4.16)	0.50	6.04	0.11	1.30	0.0003	14	W = 0	Mann-Whitney
Ventricle volume (Log transformed)								
Genotype					<0.0001	1	f = 47.04	Two-way ANOVA
Sex					NS	1	f = 1.64	
Genotype:Sex					NS	1	f = 0.523	
Brain volume (AP-0.75 to -1.75)	40.05	33.45	0.92	1.16	0.0005	14	t = 4.46	Independent t-test
Dorsal hippocampus volume	6.71	5.22	0.19	0.34	0.0057	14	t = 3.83	Independent t-test
NeuN positive neuron count								
CA1 neurons (avg per FOV)	126.1	97.0	2.8	3.6	<0.0001	15	t = 5.92	Independent t-test
CA1 neurons (avg per section)	525.8	338.8	23.0	25.0	<0.0001	15	t = 5.52	Independent t-test
CA2 neurons (avg per FOV)	97.2	66.7	3.1	4.5	<0.0001	15	t = 5.43	Independent t-test
Iba1 positive microglia								
Subiculum microglia (avg per FOV)	62.2	271.6	3.5	30.8	0.0002	13	t = 6.76	Welch's t-test[BH]
CA1 microglia (avg per FOV)	45.0	217.8	2.8	18.7	0.0008	13	t = 9.13	Welch's t-test[BH]
Hilus microglia (avg per FOV)	56.2	202.3	6.7	17.5	0.0008	13	t = 7.81	Welch's t-test[BH]
Astrocyte immunofluorescence								
Astrocyte CA1 (total fluorescence)	85.03	205.57	4.75	13.80	<0.0001	8.6	t = 8.26	Welch's t-test[BH]
Astrocyte dentate gyrus (total fluorescence)	106.20	189.20	7.32	5.49	<0.0001	12	t = 9.07	Welch's t-test[BH]
Astrocyte CA1 (fluorescence area)	21.13	24.64	0.36	0.43	<0.0001	14	t = 6.28	Independent t-test[BH]
Astrocyte dentate gyrus (fluorescence area)	22.32	24.16	0.31	0.28	0.0007	14	t = 4.32	Independent t-test[BH]
**Behavioural testing**								
Novel object								
Open field (percent time on edge)	74.41	80.26	2.67	3.09	NS	22	t = 1.38	Independent t-test
Familiarisation - object exploration (s)	32.31	36.22	1.80	4.37	NS	22	t = 0.97	Independent t-test
Novel object location (%)	32.58	19.49	2.28	3.59	0.0037	22	t = 3.24	Independent t-test
Novel object recognition (%)	44.74	23.48	1.85	3.66	<0.0001	22	t = 5.80	Independent t-test
Morris water maze								
Morris water maze training								Mixed design ANOVA
Genotype					0.032	1	f = 5.26	
Training day					<0.0001	4	f = 11.12	
Genotype:Training day					0.034	4	f = 2.72	
Latency to platform - day 1	69.68	74.09	9.03	7.97	NS	21	t = 0.32	Independent t-test[BH]
Latency to platform - day 2	56.75	52.45	4.35	6.98	NS	21	t = 0.55	Independent t-test[BH]
Latency to platform - day 3	37.73	55.42	4.75	7.12	0.045	21	t = 2.13	Independent t-test[BH]
Latency to platform - day 4	34.59	47.97	4.28	7.49	NS	21	t = 1.68	Independent t-test[BH]
Latency to platform - day 5	30.34	62.64	2.92	7.52	0.002	21.0	t = 3.27	Independent t-test[BH]
Probe trial - latency to platform zone	24.78	36.90	5.71	6.90	NS	21	t = 1.30	Independent t-test
Entry frequency into platform zone	0.93	0.38	0.25	0.26	NS	21	t = 0.59	Independent t-test
Fear conditioning (freezing %)								
Habituation (baseline freezing)	65.25	53.08	9.81	12.36	NS	21	t = 0.77	Independent t-test
Fear conditioning - baseline	19.00	15.01	3.53	5.55	NS	21	t = 0.64	Independent t-test[BH]
Fear conditioning - tone	14.57	11.39	4.20	4.65	NS	21	t = 0.49	Independent t-test[BH]
Fear conditioning - post-shock	59.68	15.33	5.87	3.36	<0.0001	19.5	t = 6.55	Welch's t-test[BH]
Contextual recall	72.00	28.06	5.57	6.60	<0.0001	21	t = 5.03	Independent t-test
Cued recall - baseline	12.77	9.13	2.60	3.62	0.412	21	t = 0.84	Independent t-test
Cued recall - tone (avg)	75.63	23.27	5.83	9.52	0.0002	21	t = 4.98	Independent t-test[BH]

Cued recall - pause (avg)	73.60	38.32	8.47	7.94	0.009	21	t = 2.85	Independent t-test[BH]
Beam walking test								
Footslips	0.38	0.42	0.15	0.14	NS	20	t = 0.16	Independent t-test
Latency to goal box (s)	7.66	8.93	1.14	2.18	NS	20	t = 0.57	Independent t-test

	**Mean**		**SEM**		**p-value**	**Degrees of freedom**	**Test statistic**	**Statistical test used**
	**WT**	**R962**	**WT**	**R962**				
**10 month-old cohort**								
NeuN positive neuron count								
CA1 neurons (avg. per FOV)	121.2	121	2.3	3.1	NS	12	t = 0.04	Independent t-test
CA2 neurons (avg. per FOV)	96.5	94.5	2.4	5.4	NS	12	t = 0.36	Independent t-test
Iba1 positive microglia								
Subiculum microglia (avg. per FOV)	59.7	60.2	11.3	4.9	NS	8	t = 0.04	Welch's t-test[BH]
CA1 microglia (avg. per FOV)	60.8	47.5	6.6	3.7	NS	12	t = 1.76	Independent t-test[BH]
Hilus microglia (avg. per FOV)	54.3	49	9.2	12.6	NS	12	t = 0.34	Independent t-test[BH]
Astrocyte immunofluorescence								
Astrocyte CA1 (total fluorescence)	218.5	210.5	15.67	23.18	NS	13	t= 0.14	Independent t-test[BH]
Astrocyte dentate gyrus (total fluorescence)	159	161.9	15.99	12.91	NS	13	t= 0.29	Independent t-test[BH]
Astrocyte CA1 (fluorescence area)	23.58	22.98	0.30	0.31	NS	13	t= 1.37	Independent t-test[BH]
Astrocyte dentate gyrus (fluorescence area)	23.6	23.18	0.28	0.43	NS	13	t= 1.84	Independent t-test[BH]
**Behavioural testing**								
Novel object								
Open field (percent time on edge)	91.82	94.48	2.26	1.42	NS	20	t = 0.85	Independent t-test
Familiarisation - object exploration (s)	33.43	34.38	1.37	2.72	NS	20	t = 0.35	Independent t-test
Novel object location (%)	25.43	26.38	1.59	2.78	NS	20	t = 0.80	Independent t-test
Novel object recognition (%)	32.43	33.27	4.8	7.12	NS	20	t = 0.15	Independent t-test
Morris water maze								
Morris water maze training								Mixed design
Genotype					NS	1	f = 0.98	ANOVA
Training day					<0.0001	4	f = 32.91	
Genotype:Training day					NS	4	f = 0.44	
Latency to platform - day 1	67.98	62.31	10.94	11.88	NS	18	t = 0.33	Independent t-test[BH]
Latency to platform - day 2	60.59	59.64	9.83	15.68	NS	18	t = 0.05	Independent t-test[BH]
Latency to platform - day 3	41.72	34.44	8.49	7.64	NS	18	t = 0.56	Independent t-test[BH]
Latency to platform - day 4	27.28	23.24	7.06	6.65	NS	18	t = 0.37	Independent t-test[BH]
Latency to platform - day 5	31.34	20.46	6.76	5.37	NS	18	t = 1.07	Independent t-test[BH]
Probe trial - latency to platform zone	29.96	28.8	6.33	8.89	NS	18	t = 0.11	Independent t-test
Entry frequency into platform zone	1.5	1.71	0.33	0.49	NS	18	t = 0.37	Independent t-test
Fear conditioning (freezing %)								
Habituation (baseline freezing)	15.72	35.29	2.91	8.03	NS	7.6	t = 2.29	Welch's t-test[BH]
Fear conditioning - baseline	18.89	28.74	5.07	6.99	NS	12	t = 1.69	Independent t-test[BH]
Fear conditioning - tone	14.63	27.7	3.38	8.62	NS	12	t = 1.14	Independent t-test[BH]
Fear conditioning - post-shock	57.05	65.08	7.51	6.3	NS	12	t = 0.71	Independent t-test[BH]
Contextual recall	58	66.14	8.58	10.4	NS	12	t = 0.58	Independent t-test
Cued recall - baseline	11.59	15.83	2.91	1.85	NS	12	t = 1.01	Independent t-test[BH]
Cued recall - tone (avg)	52.27	54.04	6.17	14.63	NS	12	t = 0.13	Independent t-test[BH]
Cued recall - pause (avg)	13.55	14.05	2.27	4.04	NS	12	t = 0.12	Independent t-test[BH]

[BH] Bonferroni-Holm correction (multiple comparison test); average (avg); field of view (FOV); not significant (NS); seconds (s); standard error of the mean (SEM); R962-hTau (R962); wildtype (WT)

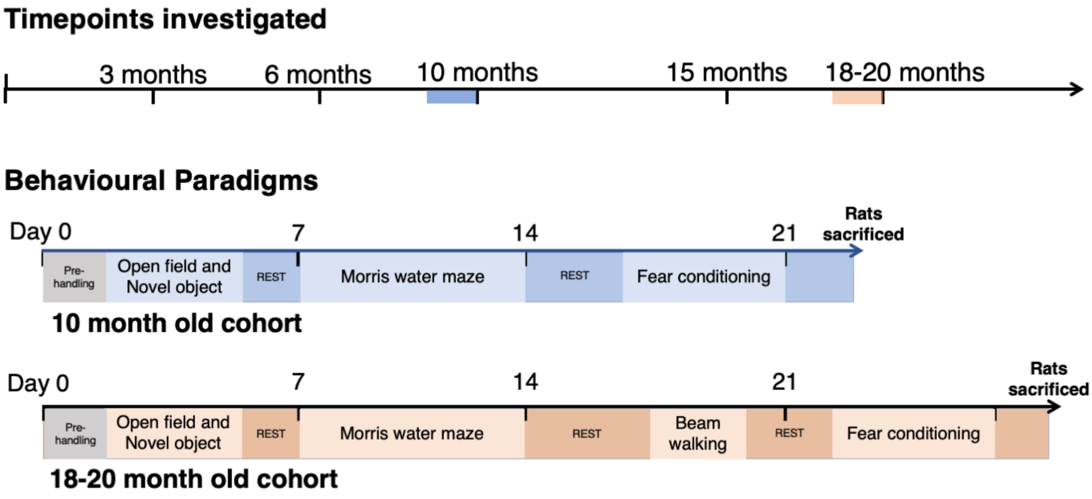

Supplementary Figure 1. *Timeline of behavioural investigations*

Behaviour was investigated in R962-hTau and WT rats, with rats sacrificed at 10 or 18-20 months after behavioural study completion. Rats were handled for at least of two days prior to behavioural testing commencing (pre-handling: day 0-2), before completing open field, novel object, Morris water maze and fear conditioning tasks. Additionally, old rats were tested for motor and coordination deficits using the beam walking test prior to fear conditioning. Rats were given 2-4 days rest between tasks. All rats were sacrificed within 4 days of behavioural testing completion.

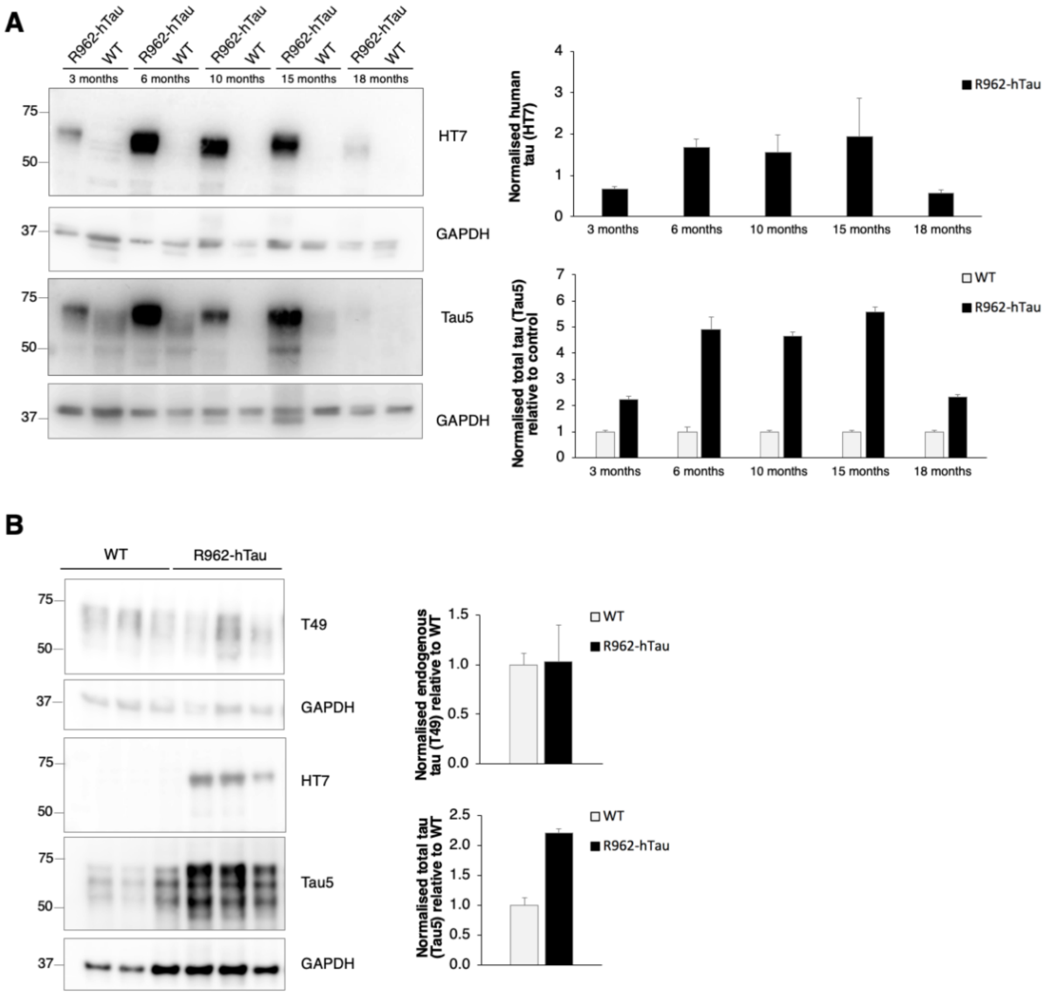

Supplementary Figure 2. *Western blotting of endogenous, human and total tau.*

(A) Western blots and quantification of human tau (HT7) and total tau (Tau5) levels in hippocampal homogenates across investigated timepoints (3, 6, 10, 15 and 18-20 months) ($n = 3$/group). (B) Western blots of 3-month-old WT control and R962-hTau whole cortex homogenates demonstrate increase in total tau levels (Tau5) is attributable to the presence of human tau (HT7) in the transgenic rats, as no obvious effect was observed on endogenous rodent tau levels (T49) between control and R962-hTau rats ($n = 3$/group). HT7, Tau5 and T49 immunoreactivity values are normalised to GAPDH levels. In addition, Tau5 and T49 levels are normalised to age-matched WT littermate levels.

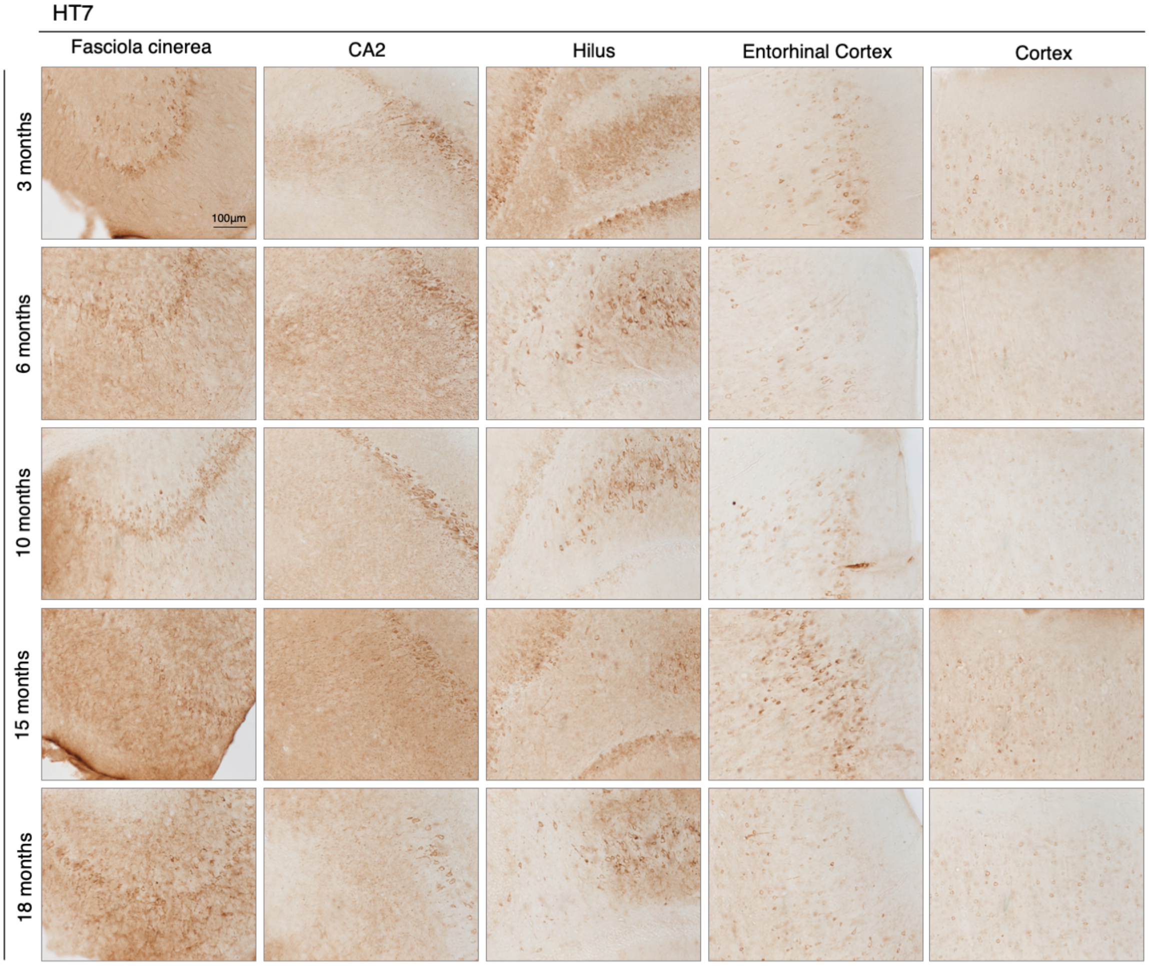

Supplementary Figure 3. *HT7-immunoreactivity progression throughout hippocampal and cortical regions*

Spatiotemporal progression of HT7-immunoreactivity through hippocampal areas (fasciola cinerea, CA2, hilus of the dentate gyrus) as well as cortical areas (lateral entorhinal cortex and upper piriform cortex, and the somatosensory cortex layers I-III) [*n* = 3-4 (3, 6, 15 months); 8-9 (10 and 18-20 months)].

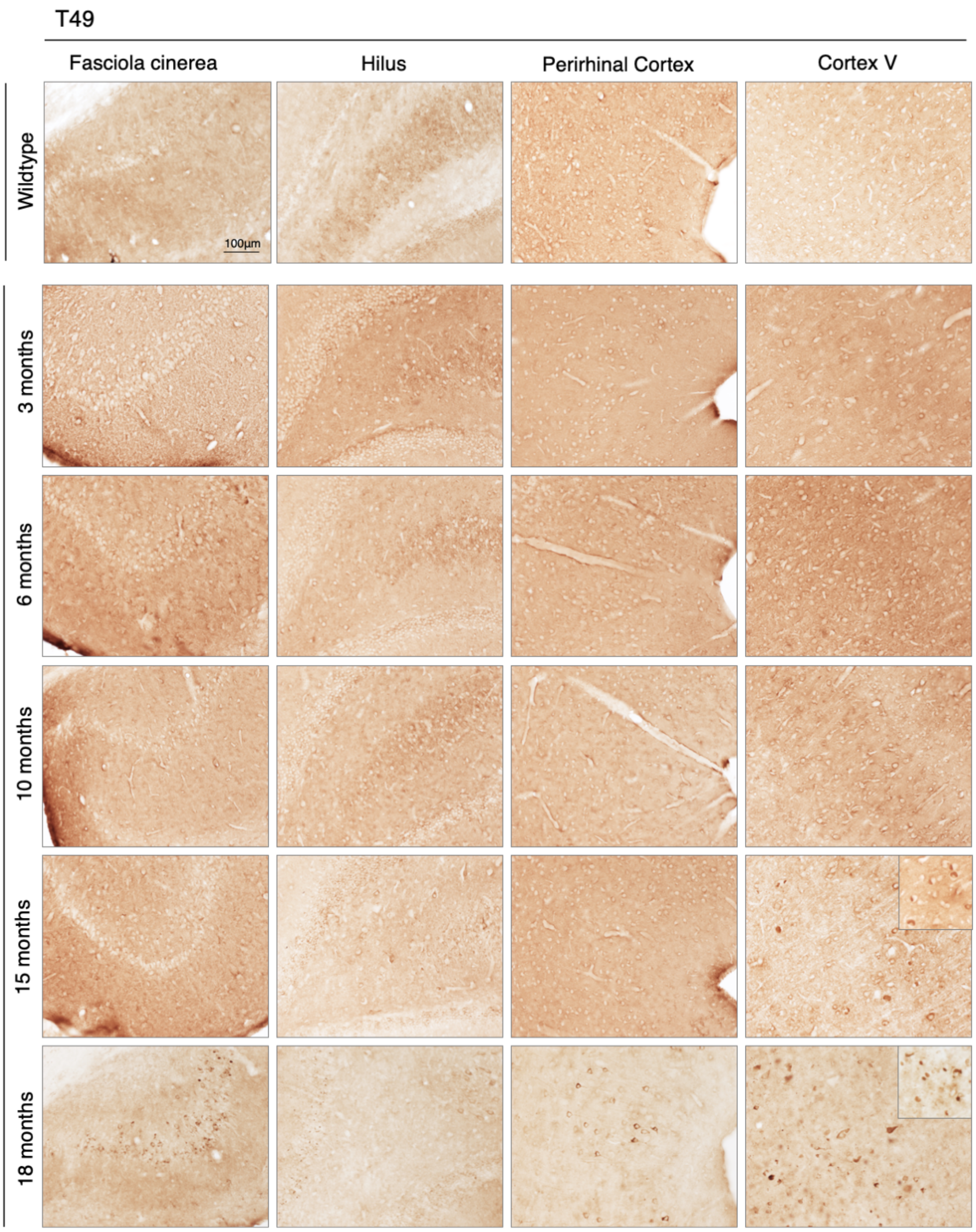

Supplementary Figure 4. *Endogenous tau recruitment throughout hippocampal and cortical regions*

Spatiotemporal progression of T49-immunoreactivity through hippocampal areas (fasciola cinerea, CA2, hilus of the dentate gyrus) as well as cortical areas (perirhinal cortex and somatosensory cortex layer V, insets layers I-III) reveals extensive endogenous tau recruitment to overt neuronal pathologies is a relatively late occurrence [*n* = 3-4 (3,6,15 months); 8-9 (10 and 18-20 months].

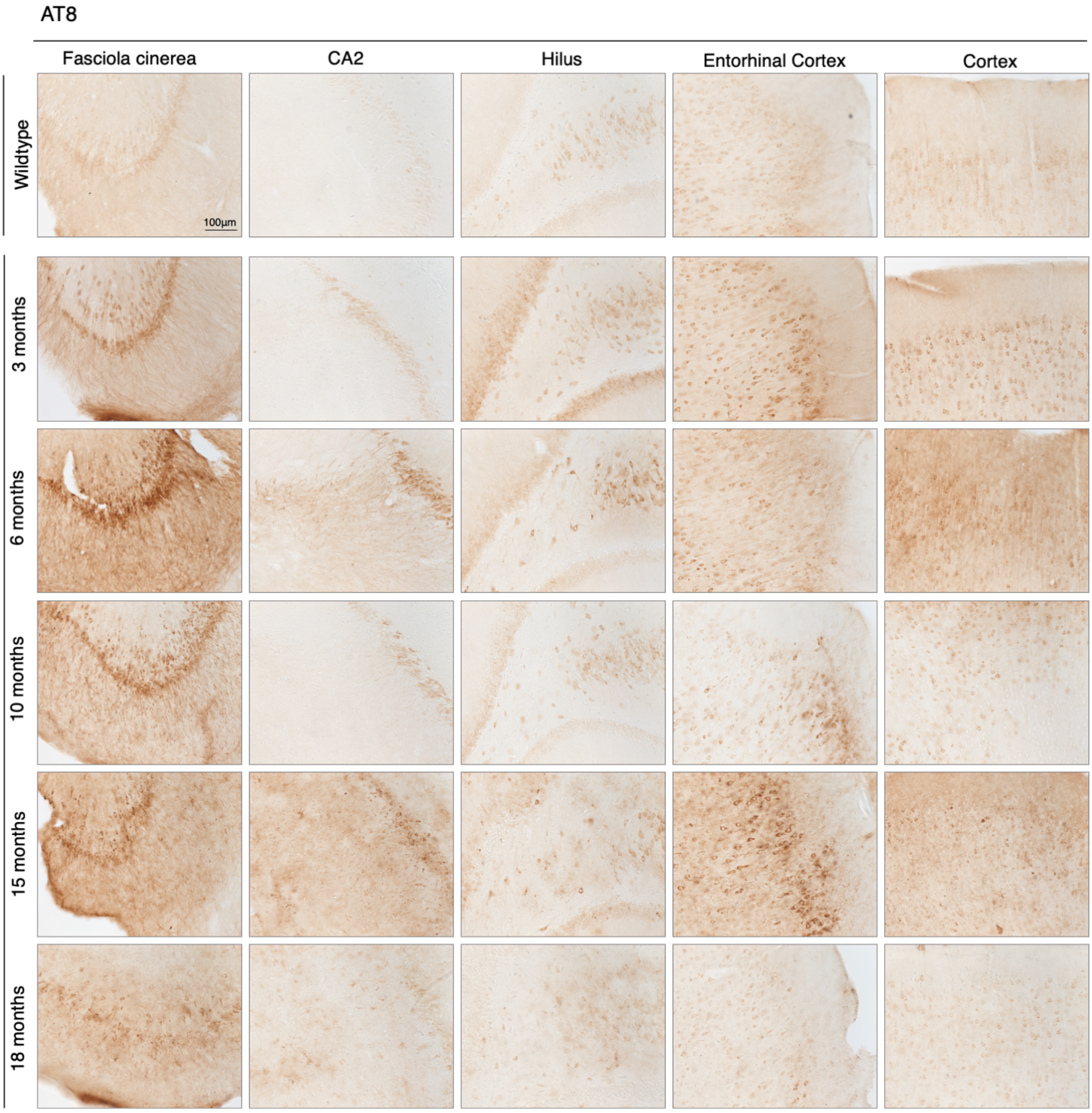

Supplementary Figure 5. *AT8-immunoreactivity progression throughout hippocampal and cortical regions*

Spatiotemporal progression of AT8-immunoreactivity through hippocampal areas (fasciola cinereal, CA2, hilus of the dentate gyrus) as well as cortical areas (lateral entorhinal cortex and upper piriform cortex, and the somatosensory cortex layers I-III) [*n* = 3-4 (3,6,15 months); 8-9 (10 and 18-20 months)].

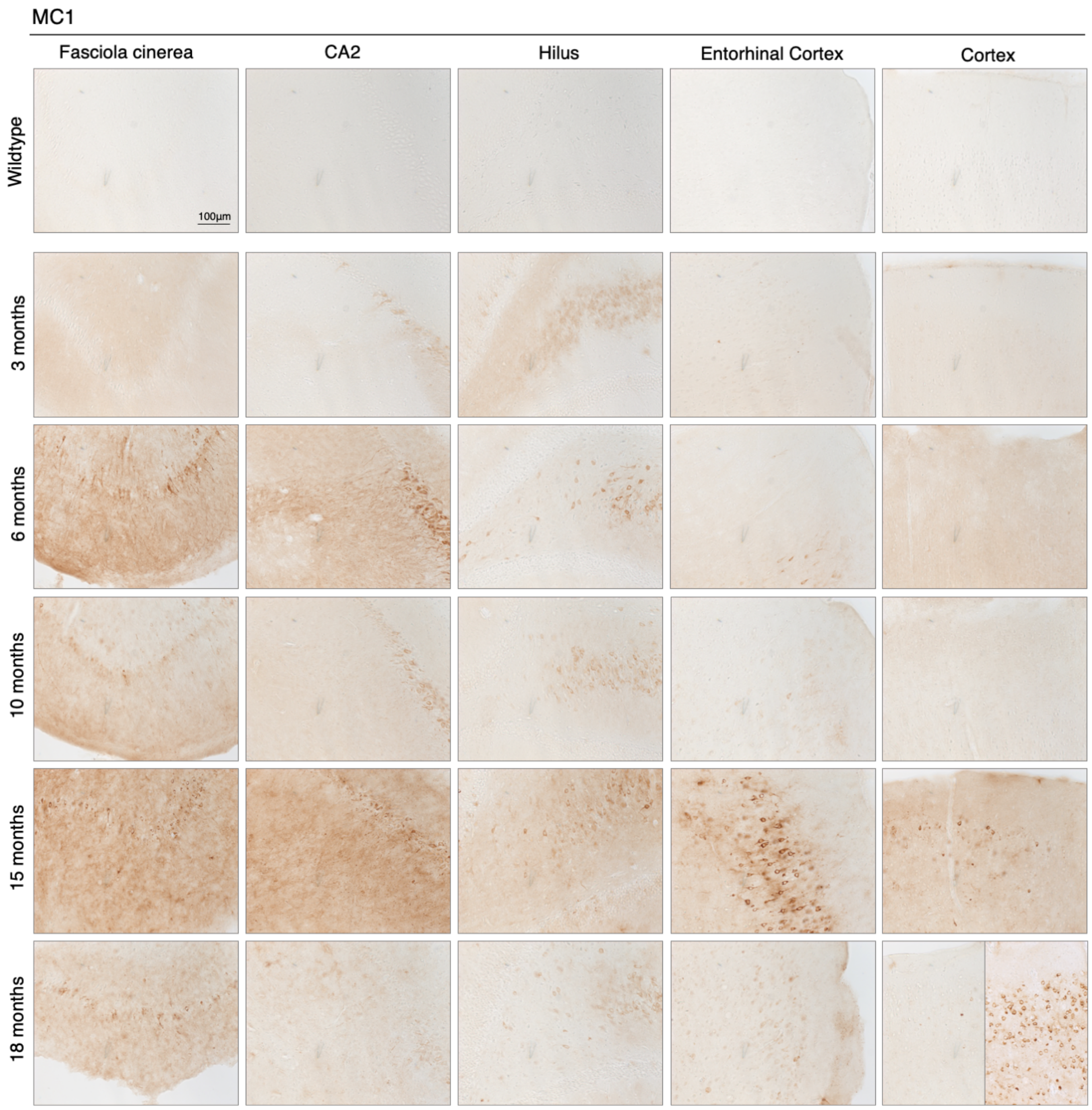

Supplementary Figure 6. *MC1-immunoreactivity progression throughout hippocampal and cortical regions*

Spatiotemporal progression of MC1-immunoreactivity through hippocampal areas (fasciola cinerea, CA2, hilus of the dentate gyrus) as well as cortical areas (lateral entorhinal cortex and upper piriform cortex, and the somatosensory cortex layers I-III) [n = 3-4 (3,6,15 months); 8-9 (10 and 18-20 months)].

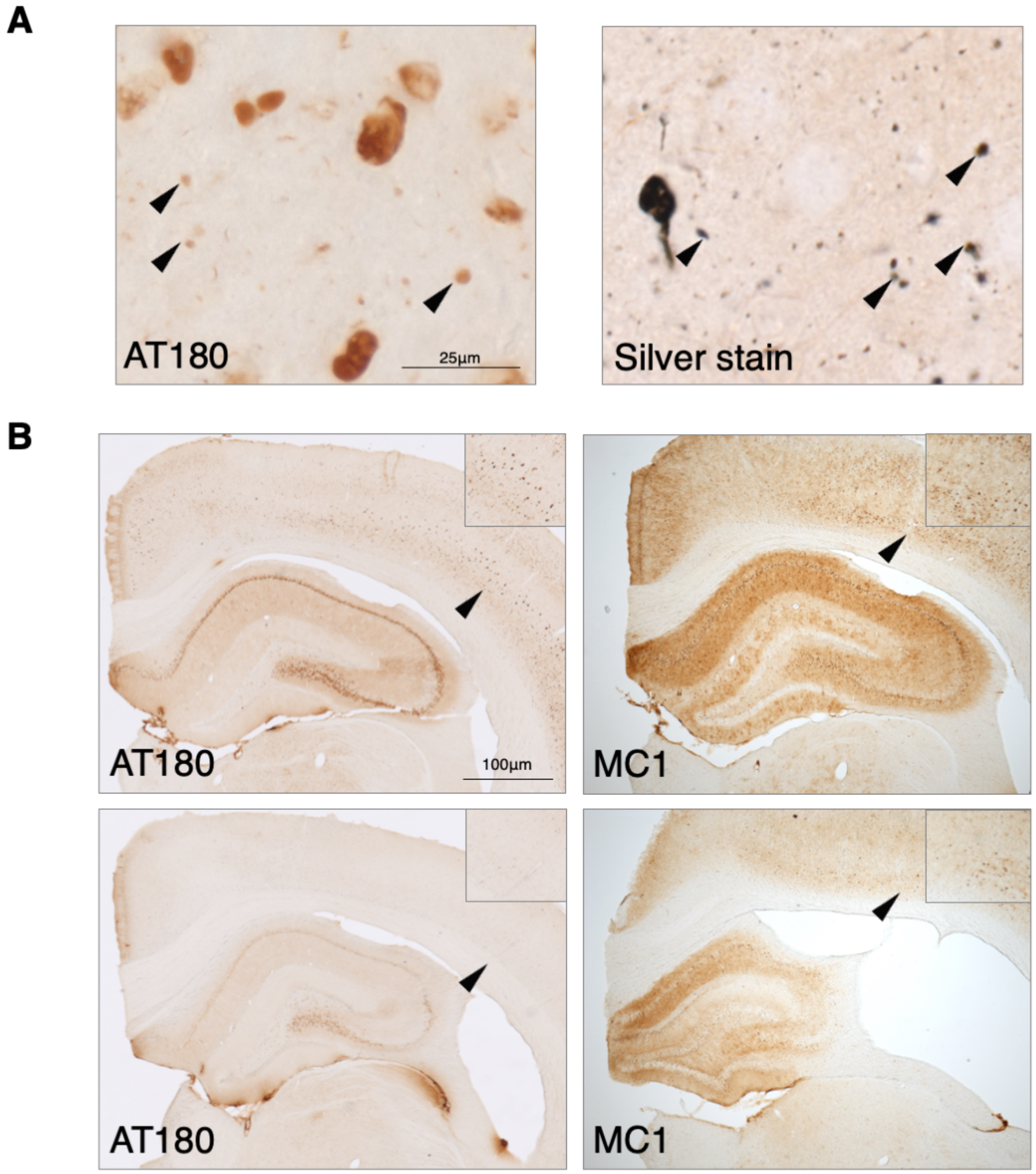

Supplementary Figure 7. *Presence of axonal spheroids and variability between staining patterns in R962-hTau rats*

(A) Presence of axonal spheroids and pick like bodies visualized using the antibody AT180 (arrows) ($n = 7$-9/group), and axonal spheroids and neuronal aggregates viewed with a Bielschowsky silver stain (arrows) ($n = 2$/group). (B) Variability between rats in tau distribution and immunoreactivity are present. At 18 months of age, one rat (top left) is seen to have numerous AT180-immunoreactivity inclusions throughout the cortex, while these are virtually absent in another rat of the same age and sex (bottom left). Similarly, differences in

MC1-immunoreactivity intensity and inclusions differ between two more examples. Extreme differences can also be observed in terms of hippocampal atrophy and lateral dilation, highlighting the diversity observed with rats with the same genetic background, carrying the same mutation. Insets 70µm across.

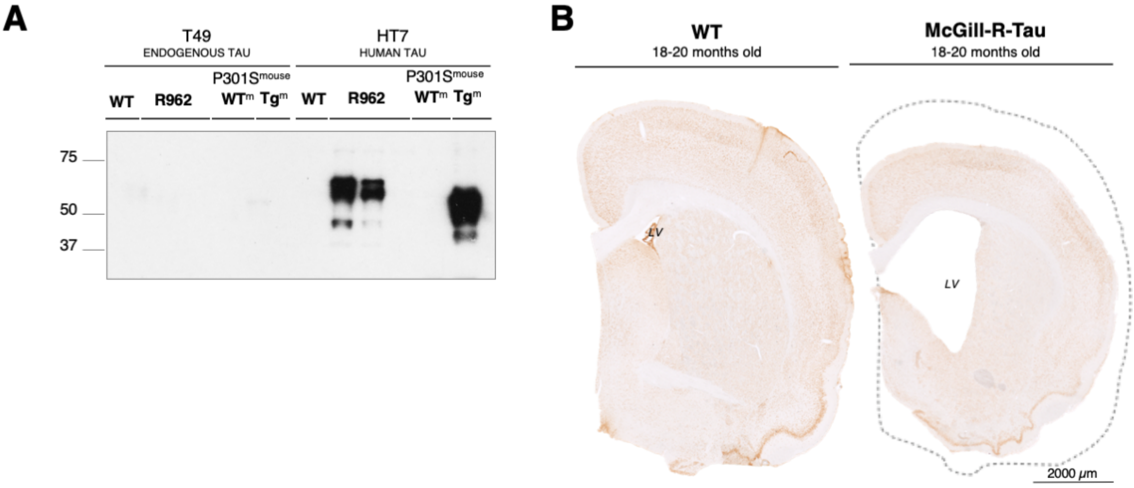

Supplementary Figure 8. *Western blotting of tau species and rostral degeneration.*

(A) 10 month old sarkosyl preparations predominately consist of human tau, with no significant endogenous tau recruitment at this time point ($n = 4$/group). (B) The rostral lateral ventricle of a 18-20-month-old R962-hTau rat with gross atrophy of cortical tissue and extensive lateral ventricle (LV) dilation compared to control ($n = 7$-9/group).

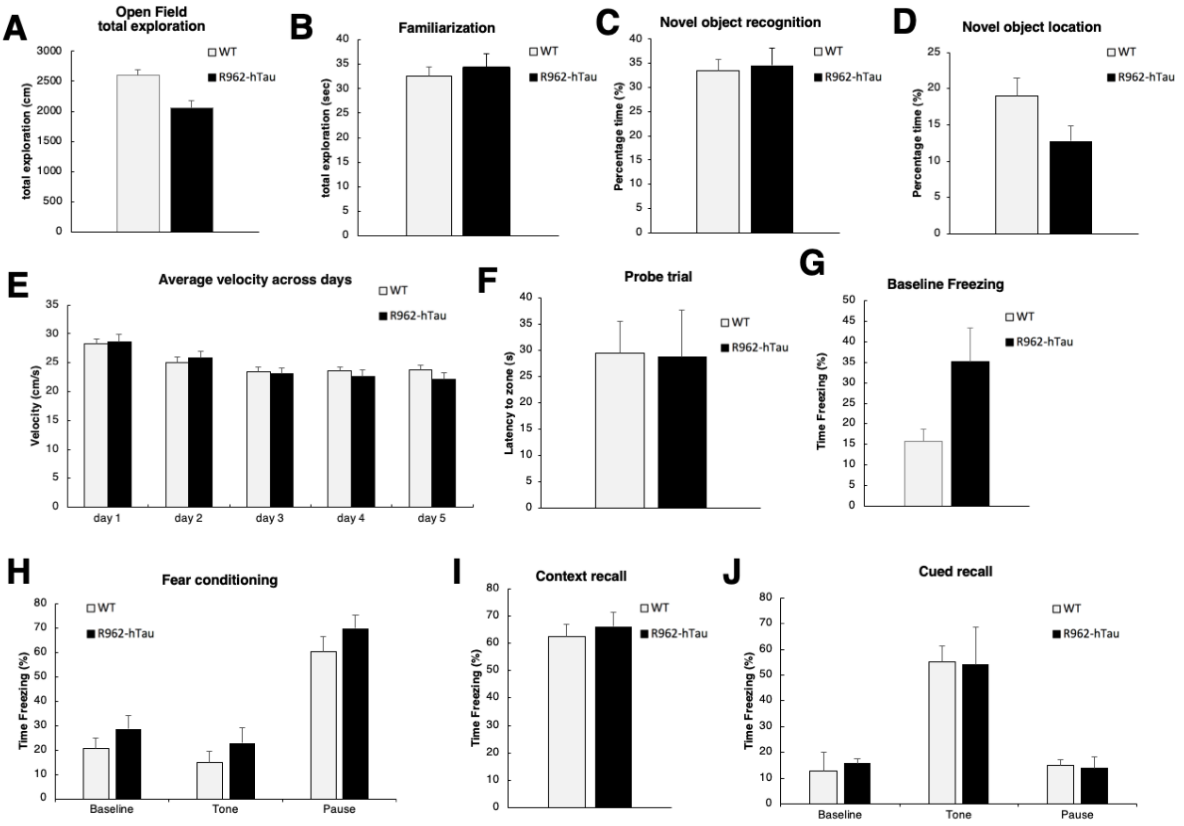

Supplementary Figure 9. *10 month old behaviour analysis in R962-hTau rats reveal no deficits*

During the open field task, total exploration was investigated (distance travelled) (A), with subsequent tests revealing no overt differences in total exploration time during familiarisation (B), novel object recognition (C) or novel object location tasks (D) at the 10 month time point (n = 14 WT; 8 R962-hTau). The Morris water maze task revealed no differences in neither swim speed between control and transgenic rats (E), nor cognitive deficits in the probe trial (F) (n = 13 WT; 8 R962-hTau). In the fear conditioning task (n = 9 WT; 5 R962-hTau), there were no differences between genotypes in time spent freezing at baseline (G) and during the conditioning (H), contextual recall (I) and cued recall phases (J).

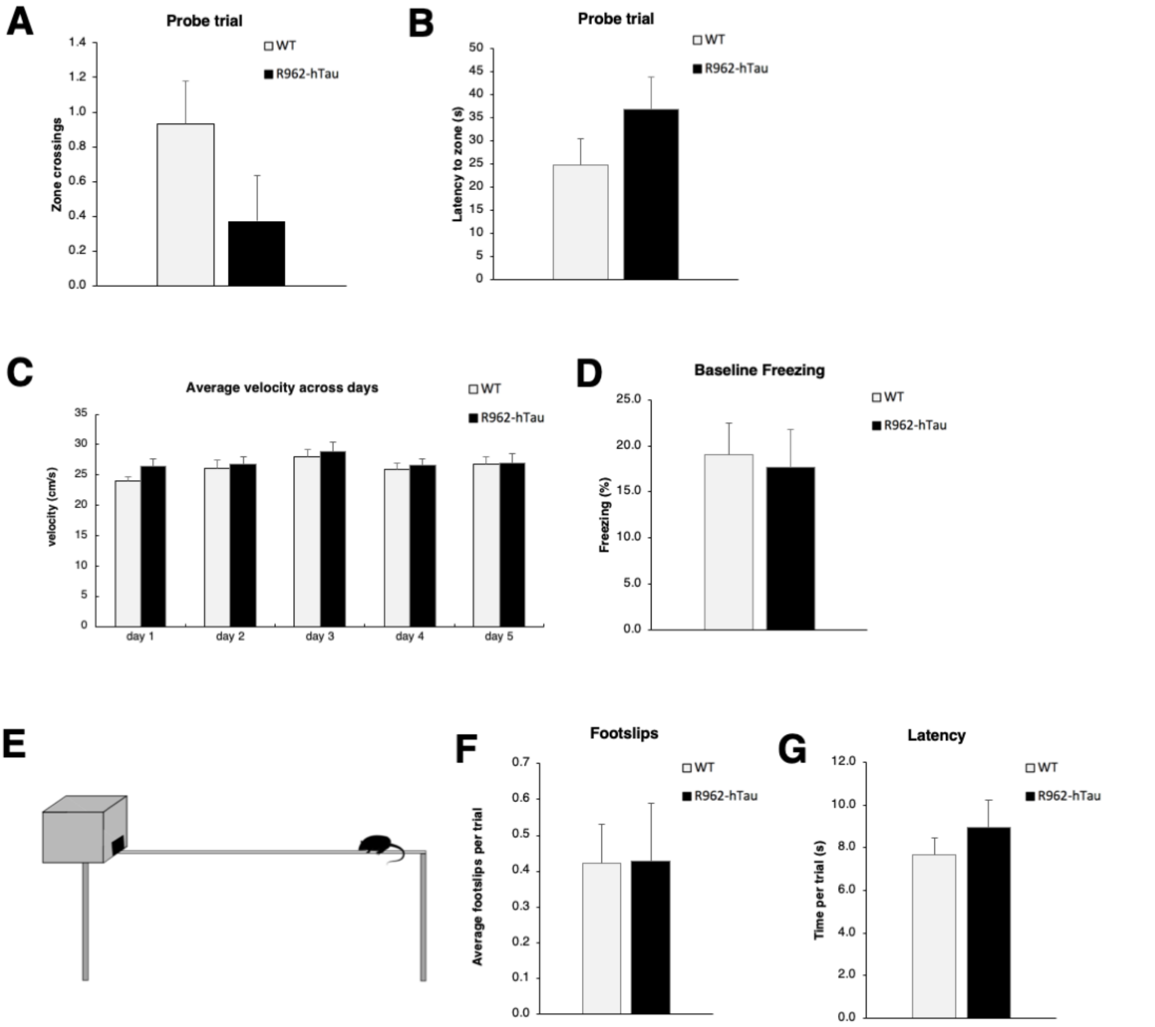

Supplementary Figure 10. *Behavioural tasks and motor coordination at 18-20-months of age*

The Morris water maze probe trial did reach a statistically difference between genotypes in terms of zone crossings (A) or latency to reach zone (B). Swimming velocity is similar in 18-20 month old R962-hTau and WT rats (C). There was no difference observed in baseline freezing during fear conditioning (D). Beam walking test (E) demonstrate no motor deficits in 18-20 months old R962-hTau rats, as determined by number of footslips (F) and latency to reach box (G) (*n* = 14-16 WT; 7-9 R962-hTau/group).

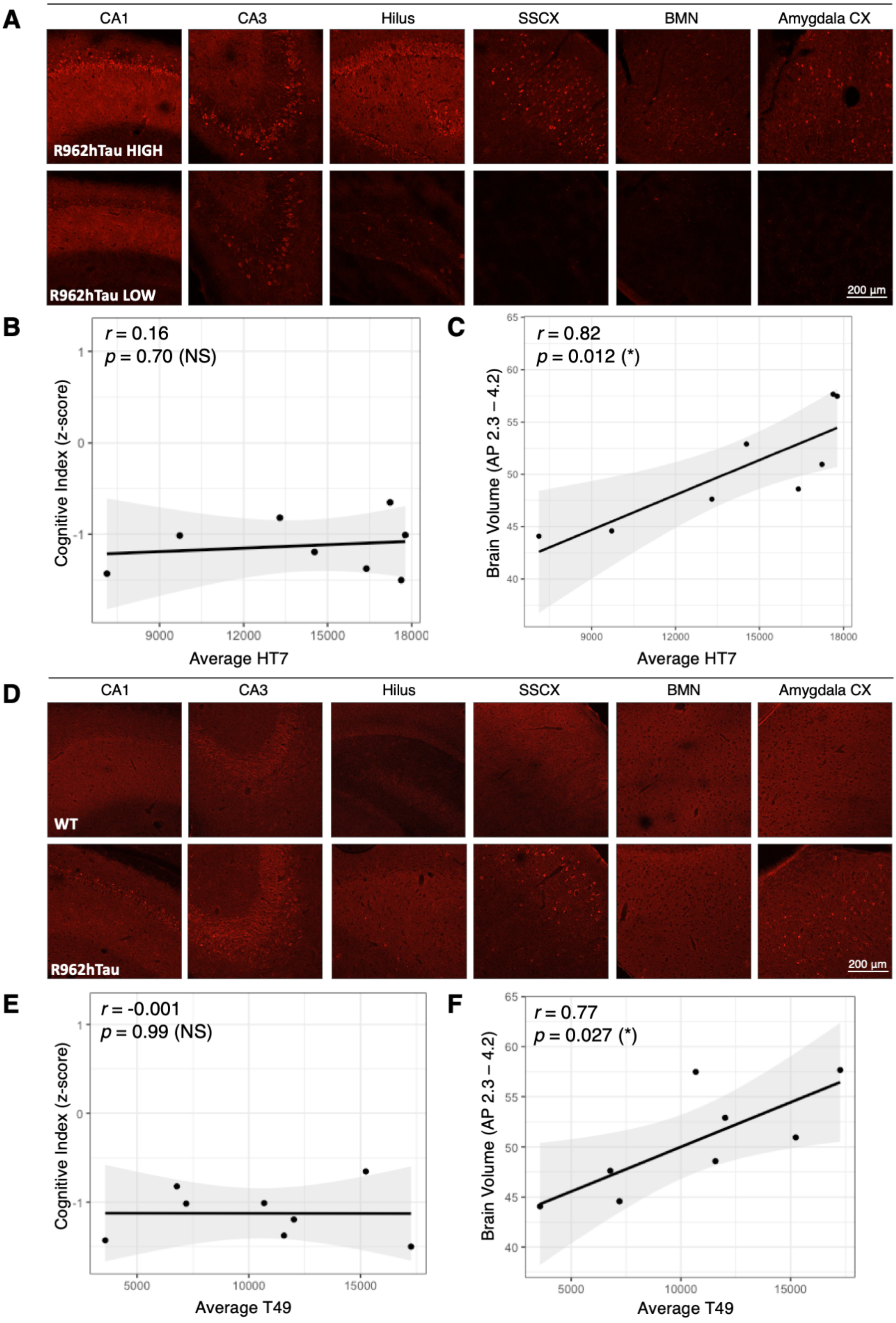
A
CA1
CA3
Hilus
SSCX
BMN
Amygdala CX
R962hTau HIGH
R962hTau LOW
200 µm
B
r = 0.16
p = 0.70 (NS)
Cognitive Index (z-score)
Average HT7
C
r = 0.82
p = 0.012 (*)
Brain Volume (AP 2.3 – 4.2)
Average HT7
D
CA1
CA3
Hilus
SSCX
BMN
Amygdala CX
WT
R962hTau
200 µm
E
r = -0.001
p = 0.99 (NS)
Cognitive Index (z-score)
Average T49
F
r = 0.77
p = 0.027 (*)
Brain Volume (AP 2.3 – 4.2)
Average T49

Supplementary Figure 11. *Correlation analysis of tau levels with neurodegeneration and behaviour*

Examples of confocal immunofluorescent images used to calculate average levels of human tau (HT7) across regions of interest [CA1, CA3, Hilus, amygdala cortex (Amygdala CX), basomedial nuclei (BMN) and somatosensory cortex (SSCX)] with rats displaying high (top) and low (bottom) levels of human tau (A). Total immunofluorescent levels of human tau, ascertained by analysis with ImageJ, did not correlate with overall cognitive performance calculated as the cognitive index *z*-score generated from behavioural tasks (B). Human tau levels positively correlate with neurodegeneration (C). Representative confocal images of endogenous tau (T49) levels from WT (top) and 962-hTau rats (D). As with human tau, average endogenous tau levels across regions of interest did not correlate with cognitive index (E), but positively correlated with regional brain volume (F). Pearson's correlation was used to analyse the association between variables. $n = 8$ R962-hTau; * $p < 0.05$, not significant (NS).
